# Novel Polymerase Gene Mutations for Human Adaptation in Clinical Isolates of Avian H5N1 Influenza Viruses

**DOI:** 10.1371/journal.ppat.1005583

**Published:** 2016-04-20

**Authors:** Yasuha Arai, Norihito Kawashita, Tomo Daidoji, Madiha S. Ibrahim, Emad M. El-Gendy, Tatsuya Takagi, Kazuo Takahashi, Yasuo Suzuki, Kazuyoshi Ikuta, Takaaki Nakaya, Tatsuo Shioda, Yohei Watanabe

**Affiliations:** 1 Department of Viral infection, Research Institute for Microbial Diseases, Osaka University, Osaka, Japan; 2 Department of Virology, Research Institute for Microbial Diseases, Osaka University, Osaka, Japan; 3 Department of Infectious Diseases, Kyoto Prefectural University of Medicine, Kyoto, Japan; 4 Graduate School of Pharmaceutical Sciences, Osaka University, Osaka, Japan; 5 Genome Information Research Center, Research Institute for Microbial Diseases, Osaka University, Osaka, Japan; 6 Department of Microbiology, Faculty of Veterinary Medicine, Damanhour University, Damanhour, Egypt; 7 Department of Laboratory Examination, International University of Health and Welfare Hospital, Tochigi, Japan; 8 Health Science Hills, College of Life and Health Sciences, Chubu University, Aichi, Japan; National Institutes of Health, UNITED STATES

## Abstract

A major determinant in the change of the avian influenza virus host range to humans is the E627K substitution in the PB2 polymerase protein. However, the polymerase activity of avian influenza viruses with a single PB2-E627K mutation is still lower than that of seasonal human influenza viruses, implying that avian viruses require polymerase mutations in addition to PB2-627K for human adaptation. Here, we used a database search of H5N1 clade 2.2.1 virus sequences with the PB2-627K mutation to identify other polymerase adaptation mutations that have been selected in infected patients. Several of the mutations identified acted cooperatively with PB2-627K to increase viral growth in human airway epithelial cells and mouse lungs. These mutations were in multiple domains of the polymerase complex other than the PB2-627 domain, highlighting a complicated avian-to-human adaptation pathway of avian influenza viruses. Thus, H5N1 viruses could rapidly acquire multiple polymerase mutations that function cooperatively with PB2-627K in infected patients for optimal human adaptation.

## Author Summary

Avian influenza (AI) virus H5N1 subtype strains have been sporadically transmitted to humans with high mortality (>60%), presenting a serious global health threat. In particular, 63% of recent human H5N1 infection cases worldwide have been reported in Egypt, which is now regarded as a hot spot for H5N1 virus evolution. H5N1 clade 2.2.1 viruses are unique to Egypt and probably have the greatest evolutionary potential for adaptation from avian to human hosts. Here, using a comprehensive database approach, we identified various novel polymerase mutations in clade 2.2.1 virus strains, isolated from patients, that enabled enhanced viral replication in both human airway epithelial cells and mouse lungs. Interestingly, the mutations identified acted cooperatively with the PB2-E627K mutation, the most well-known human adaptation mutation, to produce a further increase in viral replication in human hosts. These results provide the first broad-spectrum data on the polymerase characteristics of AI viruses that have been selected in infected patients, and also give new insight into the human adaptation mechanisms of AI viruses.

## Introduction

Influenza A viruses replicate and transcribe their genomes in the cell nucleus, catalyzed by a viral polymerase complex composed of PB2, PB1 and PA subunits. Since influenza viruses are segmented single-stranded RNA viruses with negative polarity, each of these genes is encoded in a separate viral RNA (vRNA) segment. The trimeric polymerase complex binds to vRNA and nucleoprotein (NP) to form a ribonucleoprotein (vRNP) for viral replication. The polymerase complex and viral hemagglutinin (HA), the most abundant virion surface antigen, affect the host range of influenza viruses [1–4]. The avian influenza (AI) virus HA must change its binding preference from avian–type α2,3-linked sialic acid (α2,3 Sia) to human–type α2,6-linked sialic acid (α2,6 Sia) for efficient infection in humans [4,5]. In addition, adaptation of the polymerase complex is critical for efficient viral replication in a new host [2,6]. In particular, several adaptation mutations have been identified in the PB2 gene in seasonal human influenza viruses and in AI viruses that have caused sporadic human infections. The most well-known human adaptation polymerase mutation is the PB2-E627K substitution, which is present in most seasonal human viruses, except for the 2009 pandemic H1N1 virus [7,8], and is also in some H5N1 and H7N9 human isolates and one H7N7 human isolate [7,9–11]. The PB2-E627K mutation correlates with the high virulence of H5N1 virus in mice and allows the virus to replicate efficiently in the mouse upper respiratory tract [7,12], where the temperature is slightly lower than the core body temperature. Moreover, the PB2-D701N, PB2-Q591K, PB2-K526R, PB2-G590S/Q591R and PB2-I147T/K399T/A588T mutations increase influenza virus replication in mammalian hosts [10,13–18]. Details of the mechanisms underlying mammalian adaptation due to these mutations remain largely undetermined.

Since its emergence in China in 1996, highly pathogenic AI virus subtype H5N1 has caused 842 confirmed human infections with 53% mortality (as of 23 June 2015, according to WHO; http://www.who.int/). H5N1 viruses became endemic years ago in birds in some countries; e.g., China, Vietnam, and Egypt. Continuous circulation has allowed H5N1 viruses to diverge genetically to form phylogenetically and phenotypically distinct clades (designated clades 0 to 9) in different geographic areas. In particular, clade 2.2.1 is unique to Egypt and has striking features. Clade 2.2.1 viruses typically carry mammalian-type PB2-627K [19,20], which differs from Asian H5N1 clades that generally carry PB2-627E. This implies that H5N1 viruses in Egypt should have higher polymerase activity in mammals. Since 2009, Egypt has had a higher number of human H5N1 infections than any other country, with about 63% of the cases worldwide (as of 23 June 2015, according to the WHO; http://www.who.int/). Continuous circulation of clade 2.2.1 viruses has allowed these viruses to diverge genetically to form phylogenetically distinct groups designated clade 2.2.1.1 and clade 2.2.1-B, -C and -D. Among these groups, clade 2.2.1-C has been dominant in Egypt since 2011 and was reclassified as clade 2.2.1.2 [21,22].

The majority of human isolates of AI viruses with known human adaptation mutations, as described above, carry them as a single mutation, not in combination with PB2-627K, indicating a redundancy in these mutations for human adaptation [13,16,18,23]. However, a recent study showed that PB2-K526R, particularly in combination with PB2-627K, enhanced replication of certain influenza virus subtypes [10]. Therefore, introducing additional mutations in the polymerase complex of a clade 2.2.1 virus may allow an increase in viral fitness for growth in humans. The polymerase activity of AI viruses with a single PB2-E627K mutation or a reassortment PB2 from human viruses with PB2-627K was still lower than that of seasonal human influenza viruses [24–27], supporting the above concern. Thus, investigation of adaptation mutations that clade 2.2.1 viruses may acquire in patients should provide valuable data for H5N1 pandemic risk assessment. In addition, the dynamics of clade 2.2.1 virus adaptation in patients may be a model for the evolution of increased AI virus polymerase fitness for human infections. However, information on H5N1 clade 2.2.1 virus polymerase mutations that may be selected in humans is limited.

To address this, we carried out a database search of clade 2.2.1 virus sequences that allowed a comprehensive analysis of the polymerase mutations that have been selected in clade 2.2.1 viruses isolated from Egyptian patients. Here, we identified novel polymerase mutations in clade 2.2.1 virus strains isolated from humans. Interestingly, the majority of mutations were located in the two PB1 domains that surround vRNA and act in concert with PB2-627K, possibly modifying the interaction between the vRNA promoter and the polymerase complex for optimal human adaptation. These results provide the first broad-spectrum data on polymerase characteristics that have been selected in AI viruses in infected patients and give new insight into the human adaptation strategy of AI viruses.

## Results

### Identification of human adaptation mutations in the clade 2.2.1 polymerase complex

We searched complete polymerase and nucleoprotein gene sequences (PB2, PB1, PA and NP) for amino acid mutations that presumably were selected during viral replication in H5N1-infected patients by a GISAID (http://platform.gisaid.org) database search of the sequences of human and avian H5N1 strains isolated in Egypt during 2006–2011. This yielded 61 human virus PB2, PB1, PA and NP sequences, and 91 PB2, 78 PB1, 78 PA and 81 NP avian virus sequences. At the time of this study (June 2014), the sequences of each gene searched were >70% of the all H5N1 virus sequences for that gene from H5N1 strains isolated in Egypt in the GISAID database: the remaining 30% were partial sequences and, therefore, were eliminated from this study. All sequences were obtained from isolates from clinical swabs or samples that had been passaged no more than three times in eggs and/or cultured cells (S1 Table). Since our previous study [28] indicated that clade 2.2.1 virus strains with *in vitro* adaptation mutations in eggs/cells did not become dominant in viral populations until after more than six passages, we concluded that the sequences in this study presumably represented the dominant virus populations in the infected hosts, even though this observation may not be applicable to all of the clade 2.2.1 virus isolation cases.

**Fig 1:**
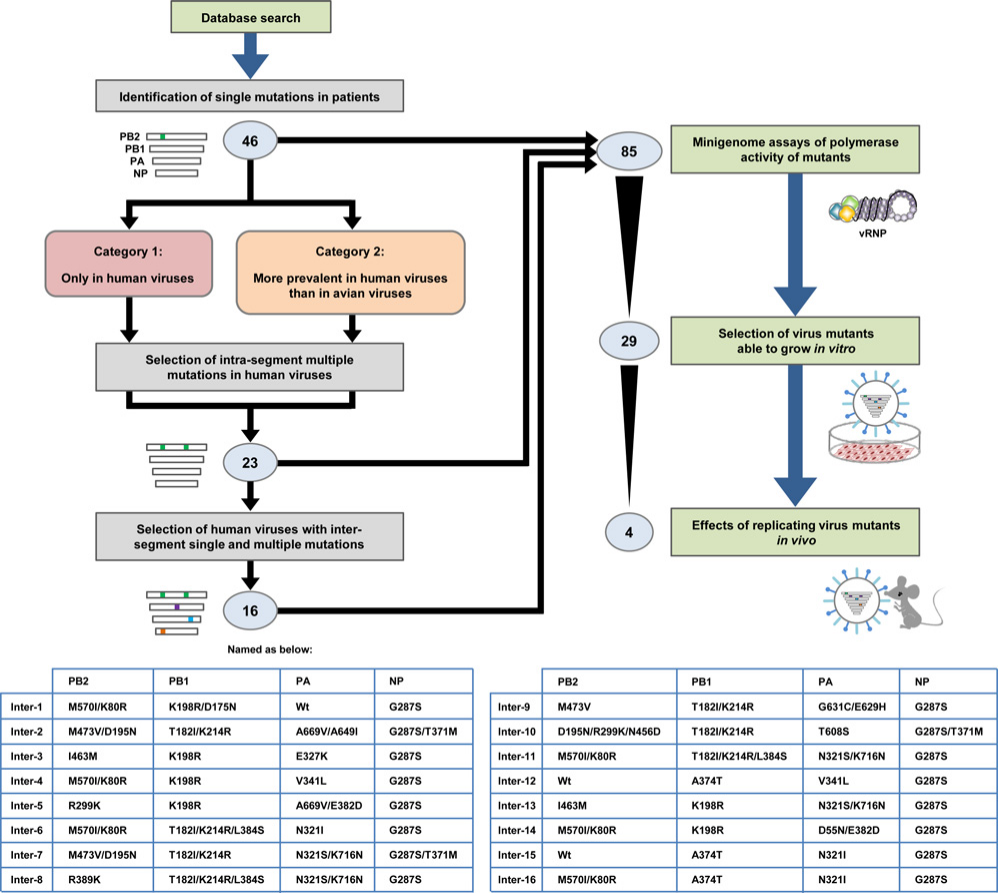
Schematic overview of selection and characterization of polymerase and NP mutations. Based on a database search of clade 2.2.1 virus sequences, we selected a total of 46 single mutations that were detected either only in human viruses (Category 1) or were more prevalent in human viruses than in bird viruses (Category 2). Since some viruses contained multiple mutations, we examined the sequences in the two virus categories for intra-segment multiple mutations and selected 23 viruses with intra-segment multiple mutations. These single and intra-segment multiple mutations were also detected in inter-segment combinations of mutations in both Category 1 and Category 2 viruses. Therefore, we searched for inter-segment combinations of single and intra-segment multiple mutations from natural human isolates, and 16 inter-segment combinations of mutations in viruses from patients were selected and designated Inter-1 to Inter-16. A total of 85 single and multiple mutations, that were carried with PB2-627K in clade 2.2.1 isolates, were assayed for polymerase activity by minigenome assays. Of these 85 mutations, 29 were introduced into EG/D1/clade 2.2.1 (wt) viruses and their effect on progeny vRNA production in human cells was investigated. From the *in vitro* progeny vRNA production results, 4 virus mutants were selected and tested for virulence in mice.

The consensus sequences of the polymerase and NP genes in H5N1 viruses isolated in Egypt were determined by aligning all of the sequence data of the vRNA segments carrying these genes. Mutations in the polymerase and NP gene sequences of the human and avian viruses were identified by comparing each gene sequence in these strains to the consensus sequence. Based on their prevalence in human and avian viruses, we identified a total of 46 single mutations (14 in PB2, 13 in PB1, 17 in PA and 2 in NP) that were either only in human viruses (Category 1) or were more prevalent in human viruses than in avian viruses (Category 2) as candidate human adaptation mutations in clade 2.2.1 viruses (Fig 1; detail shown in S2 Table). Some mutations were the only mutation in a vRNA segment in some virus strains, and some mutations were present with one or more other mutations in a vRNA segment in other virus strains (S1 Table). Therefore, we searched for multiple intra-segment mutations in viruses isolated from patients. Mutations in Category 1 viruses were thought to have been selected during viral replication in infected patients, and mutations in Category 2 viruses may have emerged in birds and then been transmitted and selected in humans with high efficacy. This implied different selective pressures for mutations in the two virus categories, with mutations in the same category perhaps having cooperative effects. Therefore, we searched for multiple intra-segment mutations in viruses in the two categories and identified 23 multiple intra-segment mutations. Single and multiple intra-segment mutations were also found in inter-segment combinations of mutations in human viruses. Therefore, we selected 16 inter-segment combinations of intra-segment mutations that were in viruses isolated from patients for further study. These inter-segment combinations of mutations were designated Inter-1 to Inter-16 (Fig 1). In all, 85 single and multiple mutations, that were present with PB2-627K in natural clade 2.2.1 isolates, were investigated in this study. The phenotypes of the 85 mutants determined in the various analyses in this study are summarized in S3 Table.

### Effect of polymerase mutations on viral polymerase activity

To investigate the effect(s) of the mutations on viral polymerase activity, we carried out minigenome assays of the polymerases with these mutations in the genetic background of influenza virus A/duck/Egypt/D1Br/2007 (EG/D1) [29,30], since EG/D1 is a representative H5N1 clade 2.2.1 strain carrying PB2-E627K. Human embryonic kidney (293T) cells and quail-origin fibroblast (QT-6) cells were used in this study. For 293T cells, polymerase activity at both 33 and 37°C (the temperatures of the human upper and lower respiratory tract, respectively) was assayed in vRNA-oriented and cRNA-oriented minigenome assays. While mRNA can be transcribed directly from vRNA-like molecules in the vRNA-oriented minigenome assay, replication to a vRNA-like molecule must occur before transcription in the cRNA-oriented minigenome assay (S1 Fig). Thus, mRNA expression in a cRNA-oriented minigenome assay is dependent on viral polymerase-mediated replication. For QT-6 cells, polymerase activity in vRNA- and cRNA-oriented minigenome assays was carried out at 37°C to allow the results to be compared with those for 293T cells at the same temperature. In this study, PB2-D701N was included as a reference mutation, although this human adaptation mutation has not yet been detected in H5N1 viruses isolated from Egyptian patients, because the known human adaptation polymerase mutations, except PB2-E627K, had not been detected in H5N1 viruses isolated from patients in Egypt at the time of this study. Preliminary experiments showed that EG/D1 polymerase had substantially higher polymerase activity than a strain with the PB2-K627E back mutation. However, the activity was still lower than that of human influenza virus A/WSN/1933 (H1N1) (S2 Fig), although this is laboratory-adapted strain. This was in agreement with previous studies of other H5N1 clade viruses [25–27], in which the polymerase activity of H5N1 viruses with a single PB2-E627K mutation was lower than that of seasonal human influenza viruses.

**Fig 2:**
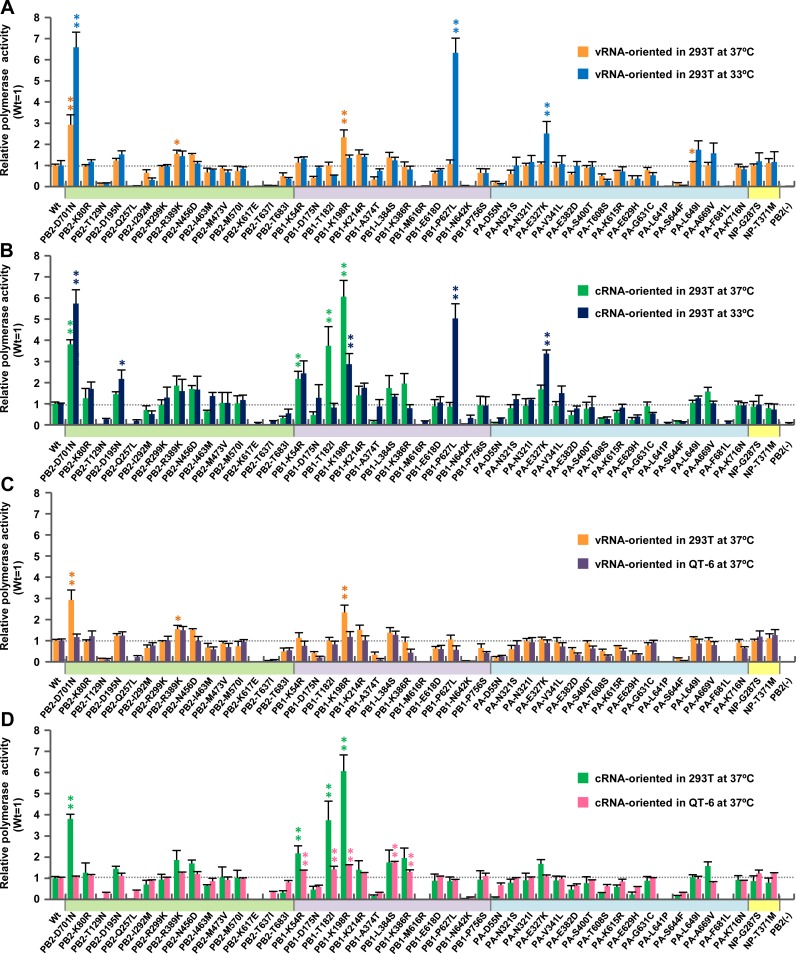
Effect of single mutations on polymerase activity. 293T and QT-6 cells were transfected with plasmids expressing EG/D1 PB2, PB1, PA or NP with the indicated single mutations, a human or chicken polymerase I-driven plasmid expressing a vRNA-oriented or cRNA-oriented luciferase reporter gene, and a *Renilla* luciferase-expressing plasmid as an internal control. After 48 h incubation at 33 or 37°C, luciferase activities were measured and normalized to the internal *Renilla* luciferase activity. The data were expressed relative to the results for EG/D1 (wt). (A) Comparison of luciferase activity at 37 and 33°C in vRNA-oriented minigenome assays in 293T cells. (B) Comparison of polymerase activity at 37 and 33°C in cRNA-oriented minigenome assays in 293T cells. (C) Comparison of polymerase activity in 293T and QT6 cells in vRNA-oriented minigenome assays at 37°C. (D) Comparison of polymerase activity in 293T and QT6 cells in cRNA-oriented minigenome assays at 37°C. Each data point is the mean ± SD of three independent experiments. Colors on each x-axis highlight different virus gene segments. Single and double asterisks indicate a *P* value <0.05 and <0.01, respectively (ANOVA with Tukey’s multiple comparison test). Asterisks for mutations with negative effects on polymerase activity were omitted for clarity.

The minigenome assays showed that several single mutations increased EG/D1 polymerase activity under some conditions. In particular, at 33°C, PB1-P627L and PA-E327K increased polymerase activity 6.3-fold and 2.5-fold, respectively, in vRNA-oriented minigenome assays (Fig 2A) and 5.0-fold and 3.4-fold, respectively, in cRNA-oriented minigenome assays (Fig 2B). In addition, in cRNA-oriented minigenome assays, PB1-T182I and PB1-K198R increased polymerase activity 3.7-fold and 6.0-fold, respectively, at 37°C and PB1-K198R also increased polymerase activity 2.9-fold at 33°C (Fig 2B).

**Fig 3:**
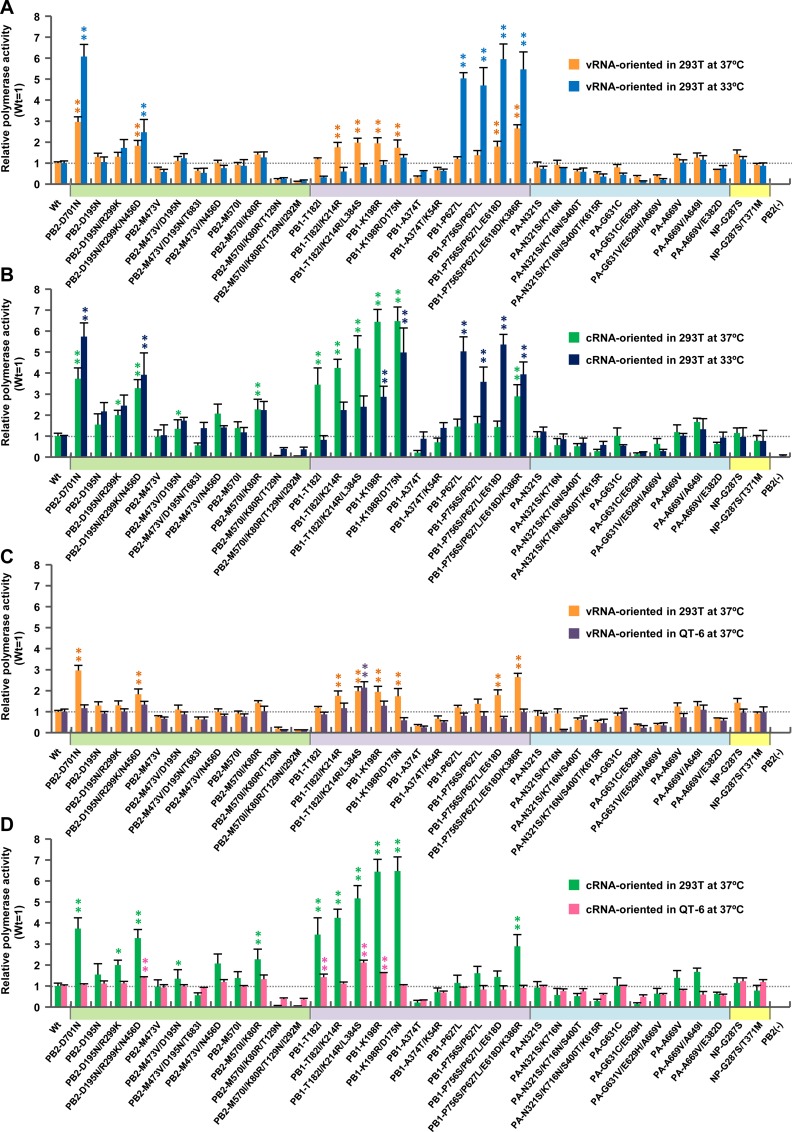
Effect of multiple mutations on polymerase activity. 293T and QT-6 cells were transfected with plasmids expressing EG/D1 PB2, PB1, PA and NP with the indicated multiple mutations, a human or chicken polymerase I-driven plasmid expressing a vRNA-oriented or cRNA-oriented luciferase reporter gene, and a *Renilla* luciferase-expressing plasmid as an internal control. Luciferase activities were assayed 48 h post-transfection and normalized to the internal *Renilla* luciferase activity. The data were expressed relative to the results for EG/D1 (wt). (A) Comparison of luciferase activity at 37 and 33°C in vRNA-oriented minigenome assays in 293T cells. (B) Comparison of polymerase activity at 37 and 33°C in cRNA-oriented minigenome assays in 293T cells. (C) Comparison of polymerase activity in 293T and QT6 cells in vRNA-oriented minigenome assays at 37°C. (D) Comparison of polymerase activity in 293T and QT6 cells in cRNA-oriented minigenome assays at 37°C. Each data point is the mean ± SD of three independent experiments. Colors on each x-axis highlight different virus gene segments. Single and double asterisks indicate a *P* value <0.05 and <0.01, respectively (ANOVA with Tukey’s multiple comparison test). Asterisks for mutations with negative effects on polymerase activity were omitted for clarity.

When multiple mutations were introduced into one of the EG/D1 vRNP proteins, an increase in polymerase activity due to the mutations acting cooperatively was observed (Fig 3A and 3B). In particular, in cRNA-oriented minigenome assays, the PB1-T182I/K214R/L384S triple mutation induced up to 5-fold and 2.4-fold greater polymerase activity than wild-type in an additive manner at 37°C and 33°C, respectively; i.e., PB1-T182I < PB1-T182I/K214R/L384S, *P* <0.05 by ANOVA with Tukey’s multiple comparison test (Fig 3B). In addition, at 33°C, the PB1-K198R/D175N double mutation had a synergistic effect and induced up to 5-fold greater polymerase activity in cRNA-oriented minigenome assays than wild-type; i.e., PB1-K198R < PB1-K198R/D175N, *P* <0.01 by ANOVA with Tukey’s multiple comparison test. The effects of these single/multiple mutations in increasing polymerase activity were comparable to that of PB2-D701N in each of the conditions studied (Figs 2 and 3).

We further investigated the effect of inter-segment combinations of mutations on polymerase activity. The results showed that inter-segment combinations of mutations had a minimal synergistic effect on increasing polymerase activity (S3A and S3B Fig). Furthermore, these mutations increased polymerase activity in a human cell-specific manner in both vRNA- and cRNA-oriented minigenome assays, although relatively moderate effects were observed in vRNA-oriented minigenome assays (Figs 2C, 2D, 3C, 3D, S3C and S3D and S3 Table), indicating that they had better fitness in human cells. Taken together, these results showed that mutations selected by viral growth in patients contributed to increased polymerase activity of clade 2.2.1 viruses in human cells at the lower temperature in these studies (33°C) and in these cRNA-oriented minigenome assays.

### Effects of polymerase mutations on viral growth in human airway epithelial cells

We next investigated the effect(s) of the polymerase mutations on the growth of clade 2.2.1 viruses in human cells using recombinant viruses generated by reverse genetics in the EG/D1 genetic background. For these studies, we selected the 8 inter-segment combinations of mutations with the greatest increase in polymerase activity in minigenome assays (i.e., Inter-1 to Inter-8, S3A and S3B Fig) and 17 of the single/multiple intra-segment mutations in the 8 inter-segment combinations of mutations. The 17 single/multiple intra-segment mutations had a range of effects on polymerase activity in the minigenome assays, from a >5-fold increase to an activity less than wild-type (Figs 2 and 3 and S3 Table). We also selected 4 single/multiple mutations with large increases in polymerase activity (>3-fold), although these mutations were not in the 8 inter-segment combinations of mutations. Thus, a total 29 single/multiple mutations and inter-segment combinations of mutations were selected for further characterization and for the rescue of replicating viruses.

**Fig 4:**
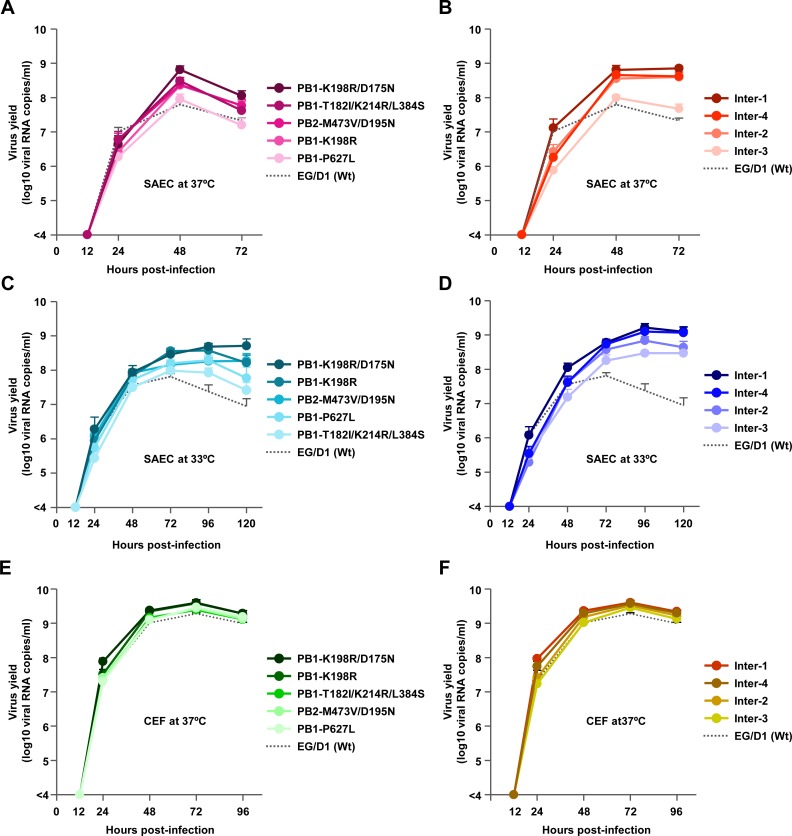
Effect of polymerase mutations on progeny vRNA production in primary human airway cells. (A−D) SAE cells were infected with the indicated viruses at an MOI of 0.03 and incubated at 37°C (A and B) or 33°C (C and D). (E−F) CEFs were infected with the indicated viruses at an MOI of 0.005 and incubated at 37°C. The culture supernatants were harvested at the indicated times and assayed by quantitative real-time RT-PCR to determine the amount of progeny vRNA. Each data point is the mean ± SD of the log10 number of vRNA copies/ml from three separate experiments.

Primary human small airway epithelial (SAE) cells, which are anatomically close to the alveoli that are an initial target for H5N1 infection in humans, were infected with one of the mutant viruses or with EG/D1 (wt) at a multiplicity of infection (MOI) of 0.03, incubated at 33 or 37°C, and progeny vRNA production was assayed to 120 h post-infection. The progeny vRNA production of the virus strains in this study are shown in S4 Fig: the data for strains that are discussed together below have been graphed together in Fig 4. Progeny vRNA production of several polymerase mutant viruses was generally higher than that of EG/D1 (wt) at later times post-infection (Fig 4). At 37°C, the PB2-M473V/D195N, PB1-K198R, PB1-K198R/D175N, PB1-T182I/K214R/L384S, Inter-1, Inter-2 and Inter-4 mutants and EG/D1 (wt) produced similar amounts of progeny vRNA at 24 and 48 h post-infection, and up to 11.0-fold more progeny vRNA than EG/D1 (wt) virus at 72 h post-infection (Fig 4A and 4B). However, the effects at later times post-infection at 33°C were more dramatic: despite similar vRNA production kinetics of the mutant viruses and EG/D1 (wt) at 24 and 48 h post-infection, the mutant viruses produced up to 25.4-fold more progeny vRNA than EG/D1 (wt) at 72, 96 and 120 h post-infection (Fig 4C and 4D). The PB1-P627L and Inter-3 mutants only produced more progeny vRNA than EG/D1 (wt) at 33°C, indicating a lower temperature-specific increase in virus replication. The post-infection times at which progeny vRNA production of the mutants peaked were different than those of EG/D1 (wt), particularly at 33°C. While EG/D1 (wt) progeny vRNA production peaked at 72 h post-infection and subsequently decreased, some mutant viruses produced increasing amounts of progeny vRNA to 96–120 h post-infection. At these later times, the difference between mutant and EG/D1 (wt) vRNA production was up to 73-fold. In contrast, in chicken embryo fibroblasts (CEFs), there was little difference between mutant and EG/D1 (wt) viruses in the amount of progeny vRNA at each time point at 37°C, and the amount was only up to 2.1-fold higher for mutant viruses than for EGD1 (wt) at 72 h post-infection (Figs 4E and 4F and S5). These results suggested that the viral mutations selected in patients had a significant effect and increased clade 2.2.1 viral growth in human airway epithelial cells, but not in avian cells, indicating a role for these mutations in H5N1 virus adaptation to humans, even though progeny vRNA production might not completely reflect infectious viral titer.

Interestingly, the PB2-D701N human adaptation mutation decreased progeny vRNA production in SAE cells (S4 Fig). A similar effect was observed for virus strains with some of the mutations selected in patients (e.g., PB1-P756S/P627L/E618D/K386R, PA-E327K, Inter-5 and Inter-8), despite their impact on polymerase activity in minigenome assays. These results indicated that increased polymerase activity did not always contribute to increased viral growth in host cells, as discussed below.

### Properties of polymerase mutations that increased progeny vRNA production in human airway epithelial cells

**Fig 5:**
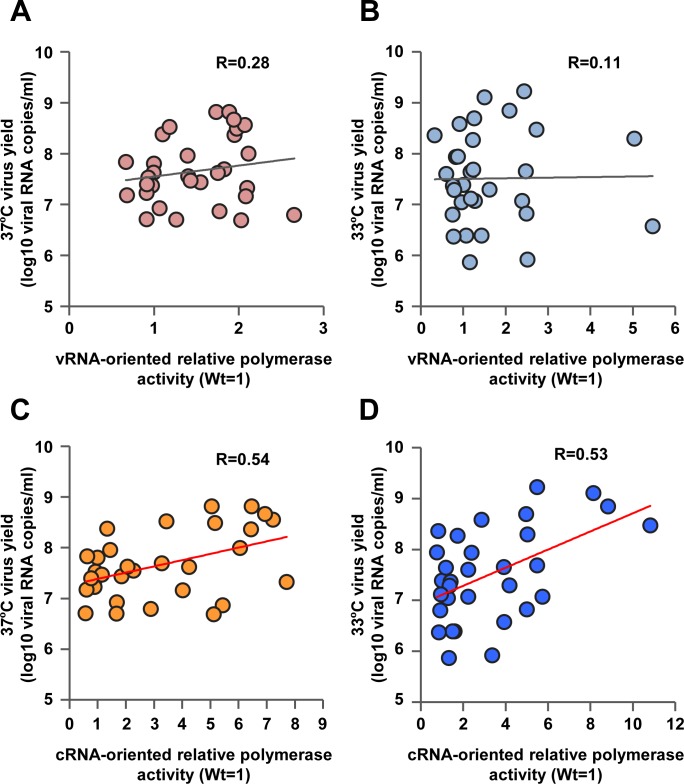
Relationship between polymerase activity and progeny vRNA production in primary human airway cells. The vRNA-oriented luciferase activity data in Figs 2A and 3A were plotted against the progeny vRNA production data in S4A and S4B Fig at 37°C (A) and 33°C (B). The cRNA-oriented luciferase activity data in Figs 2B and 3B were plotted against the progeny vRNA production data in S4A and S4B Fig at 37°C (C) and 33°C (D). The data of the 29 mutations selected to investigate viral production (Figs 3 and S4) were expressed relative to the results for EG/D1 (wt). Pearson’s correlation coefficient (R) and a linear fit of the data were also calculated.

We next investigated the relationship between polymerase properties and progeny vRNA production of the 29 virus mutant strains able to grow in human airway cells. Scatter plots (Fig 5A and 5B) showed that an increase in polymerase activity in vRNA-oriented minigenome assays was not correlated with an increase in progeny vRNA production in human airway epithelial cells (Pearson’s correlation coefficient; R = 0.28, *P* >0.05 at 37°C and R = 0.11, *P* >0.05 at 33°C). In contrast, scatter plots (Fig 5C and 5D) showed that an increase in viral polymerase activity in cRNA-oriented minigenome assays was correlated with an increase in progeny vRNA production in human airway epithelial cells, both at 37°C (R = 0.54, *P* <0.001) and 33°C (R = 0.53, *P* <0.0001). These results suggested that increased viral growth in human airway cells was not only due to an increase in mRNA synthesis (transcription) but also to an increase in both transcription and v/cRNA synthesis (replication) or to a change in the amount of transcription relative to the amount of replication.

To clarify whether increased viral growth in human airway cells was due to differences in the efficacy of virus genome replication and/or transcription, we carried out primer extension assays using a polymerase reconstitution system. In this study, 293T cells were co-transfected with plasmids expressing EG/D1 PB2, PB1, PA or NP, each carrying the indicated mutations that had a significant effect on progeny vRNA production in human airway cells, including PB2-E627K, with the PB2-K627E back mutation, and a plasmid expressing cRNA as a template for replication (cRNA to vRNA synthesis). We examined the amounts of vRNA and cRNA. As shown in S6 Fig, PB2-K627E produced less vRNA than EG/D1 (wt), indicating that PB2-E627K increased the replication activity of EG/D1 (wt) polymerase. Interestingly, polymerases containing the other mutations produced much higher amounts of vRNA than EG/D1 (wt) polymerase, indicating a greater increase in replication activity by these mutants. In addition, the effects of mutations on expression of the polymerase proteins were investigated. 293T cells were transfected with plasmids expressing PB1 or PB2 carrying the indicated mutations, which had significant effects on progeny vRNA production. At 16 h post-transfection, PB1 and PB2 proteins from cell lysates were analysed by Western blotting. Quantitative analysis showed that the mutants had no significant effect on polymerase expression levels compared to EG/D1 (wt) (S7 Fig). These results indicated that the increased replication activity of the mutants in this study was due to qualitative changes in the polymerase complex as opposed to quantitative changes, such as increased expression of mutant polymerase proteins. Taken together, these results suggested that the virus strains in this study, selected during human infections, carried polymerase mutations that increased replication and/or changed the replication/transcription activity balance in human cells and also increased virus production in human airway epithelial cells.

### Effect of polymerase mutations on viral replication in mice *in vivo*

**Fig 6:**
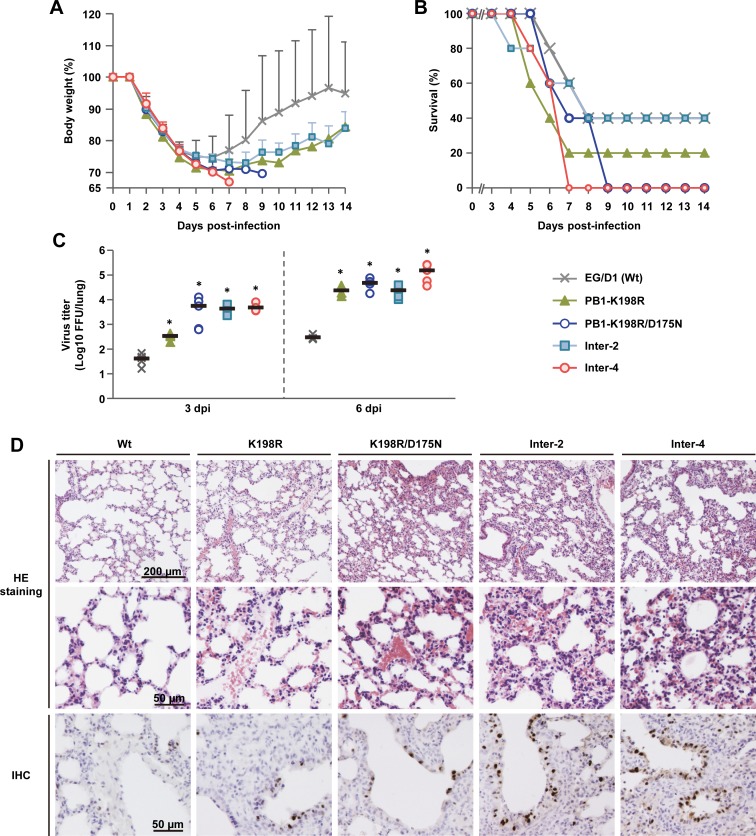
Effect of polymerase mutations on mortality and weight loss of infected mice. Five-week-old BALB/c mice were inoculated intranasally with 2.5 × 10^5^ FFU of the indicated viruses. (A) Body weight of mice (5 mice per group) infected with the indicated viruses was monitored for 14 d post-infection. The mean ± SD of the percent body weight change for each group of infected mice is shown. (B) Survival of the infected mice (5 mice per group). Mortality was calculated including mice that were sacrificed after they had lost more than 30% of their body weight. (C) Virus titers in the lungs of infected mice (5 mice per group) at 3 d (left) and 6 d (right) post-infection. Each symbol marks the titer in an individual mouse. The asterisk indicates a *P* value <0.01 (ANOVA with Tukey’s multiple comparison test). (D) Photomicrographs of hematoxylin and eosin (H&E) stained (upper and middle panels) and immunohistochemically (IHC) stained (lower panel) lung sections from mice infected with the indicated viruses at 3 d post-infection. In IHC-stained tissues, the viral antigen is stained deep brown on a hematoxylin-stained background.

To assess the relevance of the *in vitro* results of this study to *in vivo* infections, BALB/c mice were inoculated intranasally with serial dilutions of 4 virus mutants, selected because they had significant increases of both polymerase activity (Figs 2, 3 and S3 and S3 Table) and progeny vRNA production in human airway cells (Figs 4 and S4 and S3 Table) compared to ED/G1 (wt) virus, and monitored them daily for weight loss and mortality (S8 Fig). Based on these results, mice infected with 2.5 × 10^5^ focus-forming units (FFU) of the mutants showed severe weight loss with little or no recovery, and lower survival and earlier death compared to mice infected with EG/D1 (wt) (Fig 6A and 6B). We next investigated the effects of mutations on viral replication in the lungs of infected mice. Mice were inoculated intranasally with 2.5 × 10^5^ FFU of the mutants or EG/D1 (wt), and lungs were collected for virus assays at 3 and 6 d post-infection. The viral yield in lungs of mice infected with all of the mutants was >130-fold higher at 3 d post-infection and >550-fold higher at 6 d post-infection than in lungs of mice infected with EG/D1 (wt) (Fig 6C), indicating that the mutations produced several logs greater viral yields than EG/D1 (wt). This was similar to the production kinetics of the mutants in SAE cells, with much higher progeny vRNA production at later times post-infection (Fig 4A–4D).

Lungs of infected mice were examined by histopathology at 3 d post-infection. Mice infected with any of the mutants had much more severe pathological changes in their pulmonary airways and parenchymal tissues than mice infected with EG/D1 (wt) (Fig 6D upper and middle panels). Lungs of mutant-infected mice had moderate to severe bronchiolar necrosis and alveolitis with associated hyperplasia, pulmonary edema and inflammatory cell infiltrates. In contrast, lungs of mice infected with EG/D1 (wt) showed signs of limited lymphohistiocytic cell extravasation. H5 antigen was more extensively detected by immunohistochemistry in the alveolar area of lungs of mice infected with the mutants (Fig 6D lower panels). Weaker antigen staining was detected in the bronchiolar area in lungs of mice infected with EG/D1 (wt). Together, these results suggested that polymerase mutations that increased viral growth in human airway epithelial cells had a significant effect on increasing clade 2.2.1 virus growth and pathogenicity in mice *in vivo*.

### Genetic properties of influenza viruses with polymerase mutations

The phylogenetic tree reconstructed from the sequence data of H5N1 clade 2.2.1 virus PB1 genes showed an identical cluster pattern as the tree reconstructed from HA gene sequence data [21,22] (S9 Fig). Of the PB1 mutations, PB1-T182I, PB1-K214R and PB1-L384S were present in both avian and human isolates, and strains with these mutations formed two subclades distinguishable from ancestral clade 2.2.1 strains: these subclades have been designated clade 2.2.1-B and/or clade 2.2.1.2 (clade 2.2.1-C): the latter is now dominant in Egypt. This indicated that these mutations were associated with the phylogeny of clade 2.2.1-B and clade 2.2.1.2 viruses in birds. In contrast, strains with the PB1-K198R and PB1-P627L mutations were scattered or clustered in branches of human isolates and were not found in any closely related avian viruses, indicating that avian clade 2.2.1.2 viruses, some of which also carried PB1-T182I and K214R, might acquire PB1-K198R or PB1-P627L during viral growth in humans. Collectively, these results suggested that adaptation mutations in the PB1 gene emerged during the complex dynamics of H5N1 infections in birds and humans. In addition, a database search of virus gene sequences found that PB1-K198R, PB1-T182I, PB1-K214R and PB1-L384S were present in some Asian H5N1 human isolates as well as in H3N2 and H1N1 human isolates (Table 1), which also suggested a human adaptation function for these residues. The Asian human isolates carry either PB2-627E or PB2-627K, suggesting that PB1-K198R, PB1-T182I, PB1-K214R and PB1-L384S may increase Asian H5N1 virus growth by themselves and/or in combination with PB2-627K.

**Table 1:** Prevalence of PB1 human adaptation mutations in H5N1, H3N2 and H1N1 viruses.

Mutation	% of strains with mutation (no. of strains) in						
	Avian viruses		Human viruses				
	Egyptian H5N1 viruses (78)^*a*^	Asian H5N1 viruses (1449)^*a*^	Egyptian H5N1 viruses (61)^*a*^	Asian H5N1 viruses (220)^*a*^	H3N2 viruses (5592)^*b*^	H1N1 viruses (1310)^*b*^	pdm H1N1 viruses (4732)^*b*^
K198R	0 (0)	0.6 (8)	23 (14)	1.9 (4)	0.1 (8)	0.3 (5)	0.02 (1)
T182I	28.2 (22)	8.0 (116)	85.2 (52)	1.4 (3)	0.03 (2)	0 (0)	0.2 (11)
K214R	28.2 (22)	0.3 (4)	85.2 (52)	0.5 (1)	0.1 (6)	0.2 (3)	0.04 (2)
L384S	24.3 (19)	11.9 (150)	65.5 (40)	33.1 (73)	99.1 (5543)	99.8 (1308)	99.3 (4702)
P627L	0 (0)	0 (0)	11.5 (7)	0 (0)	0 (0)	0 (0)	0 (0)

^a^Polymerase sequences of H5N1 influenza viruses were obtained from the GISAID database (http://platform.gisaid.org).

^b^Polymerase sequences of H3N2, H1N1 and pdm H1N1 influenza viruses were obtained from the NCBI influenza virus resource database (http://www.ncbi.nlm.nih.gov/genomes/FLU/FLU.html).

### Effect of mutations on structural changes in the EG/D1 polymerase complex

**Fig 7:**
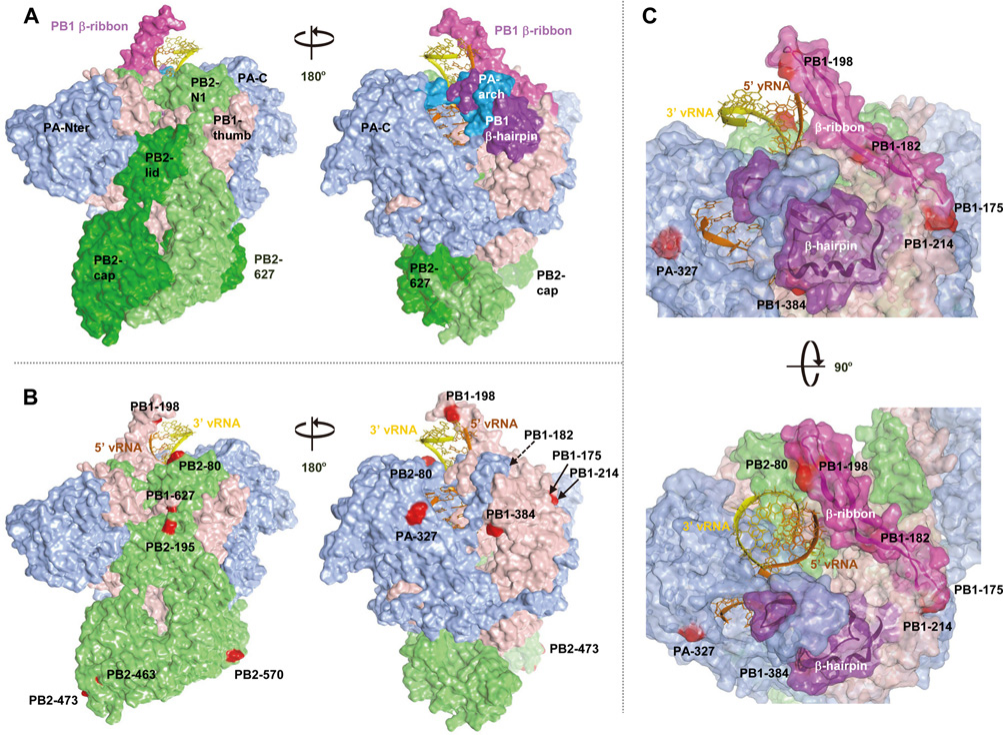
Structural model of the EG/D1 polymerase complex. Structural model of the EG/D1 heterotrimeric polymerase complex bound to the vRNA promoter. (A) Surface view of the EG/D1 structure color-coded (as described below) according to the domain structure in which the mutations identified in this study were located. Left and right structures differ by 180° in orientation. (B) Surface view of the EG/D1 structure color-coded showing PB2 (light green), PB1 (pink), PA (light blue) and the mutations in this study (red). Left and right structures differ by 180° in orientation. (C) Transparent EG/D1 surface diagram showing the mutations identified in this study (red) located mainly on the PB1 β-ribbon (magenta) and β-hairpin structures (violet) sandwiching the vRNA promoter. Upper and lower structures differ by 90° in orientation.

We generated models of the EG/D1 polymerase complex structure from a recent X-ray crystal structure of heterotrimeric polymerase from influenza A virus (Protein Data Bank ID code 4WSB) [31]. Our models showed that mutations conferring increased polymerase activity and/or progeny vRNA production in human cells were located in multiple domains of different polymerase subunits beyond the PB2-627 domain (Fig 7A and 7B) [32–35]. For PB2 mutations, K80R was in the N1-subdomain (residues 55–103) that interacts with the PB1-thumb domain, D195N was in the helical-lid domain (residues 160–212), M473V was in the Cap-binding domain (residues 318–483) and M570I was in the 627 domain (residues 538–676). For PB1 mutations, D175N, T182I, K198R and K214R were in the β-ribbon domain (residues 177–214) that interacts with vRNA, L384S was in the β-hairpin that binds to vRNA and P627L was in the thumb domain. All PA mutations were in the C-domain (residues 256–716) that may interact with the PB1-N domain. Thus, the molecular basis underlying human adaptation by H5N1 polymerase mutations appeared to be multifunctional. However, the majority of the mutations identified in this study were on the inter-subunit interfaces of PB2, PB1, PA and NP. This suggested a possible effect of the mutations in individually mediating the interaction between vRNP subunits and/or acting coordinately to optimize the vRNP structure.

Interestingly, the majority of the mutations identified in this study that showed an appreciable effect in increasing progeny vRNA production in human airway cells were clustered around the vRNA and located on two exposed PB-1 domains, the β-ribbon and the β-hairpin. An unusually long β-ribbon, where PB1-D175N, T182I, K198R and K214R were located, contacts the 3’-end of vRNA and the β-hairpin, where PB1-L384S was located, forming a 5’ vRNA binding site together with a PA-arch domain. Thus, the vRNA promoter is sandwiched in a pocket formed by the two domains (Figs 7C and S10). In addition, the β-ribbon has been reported to contact the 3’-end of the vRNA on the exterior of the polymerase complex and to have a dynamic role in relocating the vRNA into the PB1 active site during initiation of RNA synthesis [31,34]. In our simulations, a plausible model showed that mutations in the PB1 β-ribbon and β-hairpin backbone modified the topology of these two structures (S10B–S10E Fig) and changed the interaction energies between the vRNA promoter region and the known vRNA contact residues (residues 188 and 203 in the β-ribbon) [31,34] and between the vRNA promoter region and the trimeric polymerase complex (S4 Table). In particular, the PB1-T182I and PB1-K214R mutations reduced the interaction energies between the vRNA promoter and PB1 vRNA contact residues 188 and 203, but increased the interaction energies between the vRNA promoter and the trimeric polymerase complex. This implied that the vRNA contact residues may have changed in these mutant polymerases, to produce more stable conformations for interaction with the vRNA promoter. Therefore we hypothesized that the amino acid substitutions selected in human viruses may form more favorable interactions between the PB1 β-ribbon and β-hairpin and the vRNA promoter, making the polymerase complex conformation more suitable for recruiting vRNA into the polymerase complex during replication initiation. The exact mechanisms by which the polymerase mutations identified in this study affected the adaptation of avian H5N1 influenza viruses to infect humans currently remain unknown. However, our results suggested that the H5N1 clade 2.2.1 virus polymerase complex in patients has a structurally and energetically changed conformation, probably to more favorably interact with the vRNA promoter in these hosts. Although our structural study was based on a plausible model, further analyses with a better defined structural model are needed to investigate the detailed mechanism of influenza virus replication.

## Discussion

Previous studies to identify human adaptation mutations in influenza viruses have typically relied on comparison of viruses with high and low pathogenicity in mammals. However, a high-throughput screening approach would allow analysis of large numbers of mutations and identification of currently unknown human adaptation mutations. Using a comprehensive database approach in this study enabled us to identify a number of novel putative human adaptation mutations in the polymerase complex of H5N1 clade 2.2.1 viruses. These mutations acted in combination with PB2-627K and increased virus growth in both human airway epithelial cells and mouse lungs. To our knowledge, this is the first report to systematically evaluate the effects of polymerase mutations that have been selected in H5N1-infected patients on AI virus adaptation to infect humans. It should be noted that the present study was limited by the relatively small number of sequences available that were derived from human isolates of clade 2.2.1 viruses, because of the limited number of such sequences in public databases and because emergence of mutations may be stochastic and not necessarily associated with an adaptive advantage. Nonetheless, our database approach led us to identify specific mutations in the polymerase complex that have not been previously reported as human adaptation mutations, indicating the potential of the experimental approach used in this study.

H5N1 viruses are endemic in Egypt, which has a substantially higher prevalence of H5N1 viruses than any other country, with collateral human infections [20]. A previous study showed that HA mutations have been selected during viral growth in H5N1-infected patients in Egypt and have contributed to viral fitness for infecting human airway epithelia [28]. Several human clade 2.2.1 isolates that carry some of the polymerase adaptation mutations identified here also have HA mutations that increase human-receptor binding affinity. A ferret-transmission experiment showed that an H5N1 virus acquired both HA and polymerase mutations during adaptation to a new host, even though the virus used in that study was a recombinant virus intentionally carrying the well-known adaptation mutations PB2-E627K and HA-Q222L/G224S [36,37]. Collectively, these results suggested that H5N1 viruses, at least clade 2.2.1 viruses, can simultaneously acquire human adaptation mutations in multiple virus gene segments in infected patients. However, we emphasize that this study was designed to characterize polymerase mutations enabling H5N1 viruses to adapt to growth in the human airway microenvironment, not to investigate enhanced airborne transmission of H5N1 viruses in humans. Indeed, although HA mutations in H5N1 viruses isolated from patients increased virus growth in the human airway microenvironment, they also decreased HA stability as a compensatory mechanism, indicating a decrease in viral airborne transmissibility [28].

Phylogenetic analysis of the PB1, PB2, PA and NP genes in this study (S9, S11–S13 Figs) showed identical cluster patterns to that of the HA gene [21,22]. Most of the human adaptation mutations identified here were detected in H5N1 viruses in clade 2.2.1-B and in the currently dominant clade 2.2.1.2 (clade 2.2.1-C). Among these mutations, PB1-K198R, PB1-K198R/D175N, Inter-1, Inter-3 and Inter-4 were detected only in human isolates in clade 2.2.1.2, and PB1-P627L and Inter-2 were detected only in human isolates in clade 2.2.1-B. In addition, PB1-T182I/K214R/L384S and PB2-M473V/D195N were detected in both avian and human isolates in clade 2.2.1.2 and clade 2.2.1-B, respectively. All clade 2.2.1-B and clade 2.2.1.2 viruses retained PB2-627K, as observed in ancestral clade 2.2.1 viruses, and clade 2.2.1.2 viruses also carried several amino acid signature residues, including HA-128Δ/151T [21,22], which was reported to increase human-receptor binding affinity [30]. These results suggested that H5N1 viruses in Egypt probably have a greater evolutionary potential for adaptation from avian to human hosts than avian influenza viruses in other geographic areas.

Adaptation of AI virus polymerase in mammals has been studied extensively and the adaptation mutations that have been well-characterized are in the PB2 gene. PB2-627K is recognized as a dominant adaptation mutation in human influenza A viruses and facilitates efficient viral replication in mammalian cells. While several other adaptation mutations (e.g., PB2-D701N) have also been identified in PB2, the majority of these amino acid substitutions have not previously been detected together with PB2-E627K, implying that they had little or no synergistic effect with PB2-627K. In contrast, in this study we found that H5N1 clade 2.2.1 viruses that carry PB2-627K could acquire additional human adaptation polymerase mutations in infected patients, which were advantageous for viral growth in human cells and mice. It was interesting that the mutations identified here were in multiple domains of PB1, PB2, PA and NP that are remote from the PB2-627 domain (Fig 7). The effects of these adaptation mutations were cooperative with PB2-627K and increased viral growth in human cells and mouse lungs, but not in avian cells (Figs 2–6, S3–S5 and S8). Some of the adaptation mutations appreciably increased polymerase activity, especially in cRNA-oriented minigenome assays (Figs 2B, 3B and S3B and S3 Table), consistent with the changes observed in viral replication. Scatter plots also showed that increased viral replication was correlated with an increase in progeny vRNA production in human cells both at 37 and 33°C (Fig 5C and 5D). AI viruses have a diminished ability to replicate in human cells, which can be partially overcome by adaptation mutations in the viral polymerase proteins and the nuclear export protein (NEP) [1,38]. In total, these results suggested that even AI H5N1 clade 2.2.1 viruses carrying PB2-627K required other mutations for human adaptation, and that polymerase adaptation mutations selected in patients enabled an increase in viral replication for further host adaptation.

The polymerase structure models in this study indicated that the PB1-D175N, -T182I, -K198R and -K214R mutations, which had appreciable effects on H5N1 human adaptation, structurally and energetically modified the conformation of the PB1 β-ribbon that interacts with the vRNA promoter (S10 Fig). These results suggested that PB1 structural changes have an important role in influenza virus adaptation in humans, as has been previously reported [39]. Since PB1 functions as an RNA-dependent RNA polymerase, it is reasonable to hypothesize that it might undergo functional modifications during adaptation to new hosts. Amino acid substitutions in the PB1 β-ribbon and β-hairpin domains were detected in the 1918 human pandemic influenza virus when its sequence was compared to the avian influenza virus consensus sequence [40], supporting the above ideas. While transcription is performed by a *cis-*acting polymerase that forms part of the vRNP, replication is initiated by a *trans*-acting polymerase complex at the 3’-end of vRNA that is released from the resident polymerase complex [2,41]. This indicated that the mechanism underlying replication initiation was distinct from that of transcription and suggested that, at the replication initiation step, the adaptive mutations that have been identified here might influence the process by modifying vRNA packaging into the complex to form replicative vRNP. However, we cannot exclude the possibility that the mutations identified in this study affected the interaction between vRNP and host factors that mediate vRNA assembly and/or replication, as the ran binding protein 5 does [42].

In this study, polymerase activity was not always associated with viral growth in human cells and/or pathogenicity in mice. For example, PA-E327K increased polymerase activity, but had no appreciable effect on progeny vRNA production by EG/D1 (wt) in human cells. In addition, PB2-D701N caused no increase in progeny vRNA production in the EG/D1 genetic background carrying PB2-627K, in spite of the strong effect of PB2-D701N in the minigenome assay. It is possible that mutations such as PA-E327K and PB2-D701N caused the loss of some functional balance in EG/D1 vRNP carrying PB2-627K. For example, both the PB2-E627K and PB2-D701N mutations increased PB2 binding to host importin-α isoforms to indirectly regulate polymerase activity [43–45], suggesting that an excessive shift in vRNP transport between the nucleus and cytoplasm may have led to lower progeny vRNA production by the EG/D1 strain with the PB2-E627K/D701N mutations. In contrast, introduction of the PB1-K198R and PB1-T182I mutations in EG/D1, which increased progeny vRNA production in human cells, had a complementary role and increased the c/vRNA synthesis that was restricted in human host cells (Figs 2–5, S3, S6 and S7), indicating differences in their mechanism from PB2-E627K and PB2-D701N. A previous study reported that a moderate, but not excessive, increase in polymerase activity led to the greatest increase in pathogenicity in mice infected with AI viruses with adaptation mutations [14], which also supported the idea that AI viruses require a balance in polymerase functions in a new host. However, Inter-1, Inter-2 and Inter-4 mutations had a minimal synergistic effect in minigenome assays, but appreciably increased viral growth in both human cells and/or mice. It is possible that these inter-segment combinations of mutations affected steps other than RNA synthesis (e.g., nuclear export of vRNP) to synergistically enhance viral growth in mammalian hosts. Similar inconsistent effects between polymerase activity and viral growth have been reported by others [13,14,17]. Viral polymerase proteins have been suggested to co-evolve in influenza virus strains [46,47]. Taken together, these results indicated that regulation of the functions of AI adaptation genes in new hosts is complicated and polymerase complex proteins may co-evolve during influenza virus adaptation to humans with mutations that act cooperatively in the overall viral life cycle. The exact mechanism(s) by which the polymerase mutations identified in this study enable influenza virus human adaptation currently remains unknown and further analyses are needed to elucidate the underlying molecular basis of adaptation.

Several of the human adaptation polymerase mutations identified in this study (e.g., PB1-K198R and PB1-T182I) have been detected in H5N1 viruses that are circulating in different geographic areas of Asia, with either amino acid E or K at the PB2-627 residue (Table 1). Therefore, these mutations could be useful as human adaptation markers for a wide range of H5N1 viruses in the field, although not all of the adaptation mutations identified here may have the same effects in other influenza virus strains.

In conclusion, we have identified a number of putative human adaptation polymerase mutations that have not previously been reported. These mutations were widely located throughout the structural domains of the polymerase complex beyond the PB2-627 domain. The adaptation mechanisms of these mutations are probably multifactorial, highlighting the complexity of polymerase functions and of influenza virus polymerase adaptation to new hosts. Our findings also suggested that AI viruses might adapt by multiple pathways to enable their growth in humans. These data provide a novel insight into the functions of the influenza virus polymerase complex and help our understanding of the mechanism of AI virus adaptation to new hosts.

## Materials and Methods

### Ethics statement and biosecurity

All studies using anonymized primary human cells were conducted according to the principles expressed in the Declaration of Helsinki and approved by the Institutional Review Board of Osaka University (approval number 21–3). All animal experiments were conducted in compliance with Japanese legislation (Act on Welfare and Management of Animals, 1973, revised in 2012) and guidelines under the jurisdiction of the Ministry of Education, Culture, Sports, Science and Technology, Japan (Fundamental Guidelines for Proper Conduct of Animal Experiment and Related Activities in Academic Research Institutions, 2006). Animal care, housing, feeding, sampling, observation, and environment enrichment were performed in accordance with the guidelines. The protocols of animal experiments were approved by the Animal Experiment Committee of the Research Institute for Microbial Disease, Osaka University (approval number H24-07-1).

### Biosecurity and biocontainment

All experiments with live H5N1 viruses were performed at enhanced biosafety level 3+ (ABSL3+) in the Research Institute for Microbial Diseases, Osaka University, which has been approved for handling H5N1 viruses by the Ministry of Agriculture, Forestry and Fisheries of Japan. All studies with recombinant DNAs were conducted under the applicable laws in Japan and approved by the Biological Safety Committee of the Research Institute for Microbial Diseases, Osaka University (approval number 3439) after risk assessments were conducted by the Living Modified Organisms Committee of the Research Institute for Microbial Diseases, Osaka University and, when required, by the Ministry of Education, Culture, Sports, Science and Technology of Japan.

The ABSL3+ facility of the Research Institute for Microbial Diseases, Osaka University, consists of negative-pressure laboratories in which all *in vivo* and *in vitro* experimental work is carried out in class 3 isolators or class 3 biosafety cabinets, which also operate under negative pressure. Air exhausted from the class 3 units is filtered by High Efficiency Particulate Air (HEPA) filters and then leaves the facility via a second set of HEPA filters. Also, users must check pressure gauges when entering and inside the ABSL3+ facility. The ABSL3+ has a dedicated electrical generator in the event of power loss.

Only authorized personal that have received appropriate training can access the ABSL3+ facility. All activities inside the ABSL3+ laboratories are monitored via video cameras. All personnel working at the ABSL3+ facility wear a full Tyvek suit, FFP3 facemasks and multiple pairs of gloves. Furthermore, all personnel conducting this study were vaccinated against seasonal and H5N1 influenza viruses. For animal handling in the facilities, personnel always work in pairs. The facility is secured by procedures recognized as appropriate by the institutional biosafety officers at the Research Institute for Microbial Diseases, Osaka University, and by Japan government inspectors. Antiviral drugs are directly available to further mitigate risks upon incidents.

In this study, all the mutations introduced into recombinant EG/D1 virus (clade 2.2.1) were naturally detected as single/multiple intra-segment mutations or inter-segment combinations of the mutations in clade 2.2.1 viruses isolated from patients, except artificial mutation PB2-D701N. Furthermore, a mouse infection study was performed after observing no increase in progeny vRNA production of the selected viruses carrying mutations compared to previously published studies, and the PB2-D701N mutant virus was excluded from the *in vivo* mouse infection study although this mutant showed reduced progeny vRNA production in human cells compared to EG/D1 (wt) virus.

### Database search

Published complete sequences of 152 PB2, 139 PB1, 139 PA and 142 NP genes from influenza A virus subtype H5N1 strains isolated in Egypt from 2006–2011 were obtained from the GISAID database (http://platform.gisaid.org). Because there was limited availability of genetic sequences from human isolates of H5N1 viruses since 2012 (only two in 2012 and zero in 2013–2014) at the time of this study (June 2014), we focused on genetic variations of clade 2.2.1 viruses from 2006–2011. These sequences were aligned using the MAFFT program [48]. PB2 mutations in 61 human and 91 avian H5N1 virus strains, PB1 mutations in 61 human and 78 avian H5N1 virus strains, PA mutations in 61 human and 78 avian H5N1 virus strains, and NP mutations in 61 human and 81 avian H5N1 virus strains were identified by comparing the sequences in these strains to a consensus sequence of each protein that was determined from the aligned sequences of all the Egyptian H5N1 strains. The prevalence of the mutations in the human and avian virus strains was then calculated and compared between viruses isolated from the two hosts.

### Cells

293T cells, QT-6 cells and Madin-Darby canine kidney (MDCK) cells were obtained from the RIKEN BioResource Center Cell Bank (http://www.brc.riken.jp/lab/cell/english/) and maintained in Dulbecco’s modified Eagle’s medium or Ham’s F-12K medium supplemented with 10% heat-inactivated fetal calf serum (FCS) at 37°C in a humidified atmosphere of 95% air and 5% CO_2_ as previously described [30]. Primary small airway epithelial (SAE) cells (Lonza Corporation) were maintained according to the manufacturer’s recommendations. Chicken embryo fibroblasts (CEFs) were prepared from 10-day-old embryonated eggs.

### Virus preparation

Influenza viruses were grown in 10-day-old embryonated chicken eggs. The allantoic fluid was then harvested and stored at -80°C as seed viruses. For subsequent studies, allantoic fluids and culture supernatants were pre-cleared by centrifugation at 3,000 rpm for 20 min and filtration through 0.45 μm filters, and viruses were then purified by centrifugation at 25,000 rpm for 2 h through a 20–60% sucrose gradient. After collecting the virus-containing fractions, viruses were suspended in phosphate-buffered saline (PBS), layered on a 20% sucrose cushion and pelleted by centrifugation at 25,000 rpm for 2 h. Virus pellets were resuspended in PBS and aliquots were stored at −80°C as working stocks. Virus titers were assayed as FFU by focus-forming assays [30] using MDCK cells.

### Minigenome assays

293T and QT-6 cells (90% confluent in 24-well plates) were transiently transfected with the following plasmid mixture: polymerase II-driven pcXN2 plasmids each expressing the PB2, PB1, PA or NP gene of A/duck/Egypt/D1Br/2007 (EG/D1) [29,30], and a human or chicken polymerase I-driven pPol plasmid expressing either an RNA containing a negative-sense firefly luciferase reading frame bounded by the 5’- and 3’-UTRs of EG/D1 vRNA or an RNA containing a positive-sense firefly luciferase reading frame bounded by the 5’- and 3’-UTRs of EG/D1 cRNA. To monitor transfection efficiencies, cells were also transfected with a polymerase II-driven plasmid expressing *Renilla* luciferase. The total amount of plasmid mixture was 1 μg/well and the PB2:PB1:PA:NP:Pol I:*Renilla* luciferase ratio was 5:5:5:30:5:1. Cells were transfected with the plasmid mixture using *Trans*IT-LT1 (Mirus) and incubated at 33 or 37°C. At 48 h post-transfection, the cells were lysed and luciferase activity was determined by the dual-luciferase assay system (Promega) according to the manufacturer’s instructions. Luciferase activity values were normalized relative to the *Renilla* luciferase activity.

### Generation of viruses by reverse genetics

Recombinant viruses were generated with a plasmid-based reverse genetics system in the EG/D1 (wt) virus genetic background as previously described [30,49]. Mutant PB2, PB1, PA and NP genes were generated by PCR-based site-directed mutagenesis. All constructs were completely sequenced to ensure the absence of unwanted mutations. Recombinant viruses were propagated by single passage in eggs. Genes of the virus stock were sequenced to detect possible emergence of revertants during amplification.

### Progeny vRNA production in SAE cells

SAE cells and CEFs were infected in triplicate with the indicated viruses at an MOI of 0.03 and 0.005, respectively. The virus inoculum was removed after 1 h and the cells were washed with PBS, followed by further incubation at 33 or 37°C. At the indicated times post-infection, vRNA titers in the cell culture supernatants were assayed by quantitative real-time RT-PCR as described below.

### Quantitative real-time RT-PCR

Progeny vRNA was extracted from the culture medium of infected cells with a QIAamp Viral RNA Mini Kit (Qiagen) and assayed by quantitative real-time RT-PCR (QuantiTect Probe RT-PCR Kit, Qiagen) using primers targeting the M gene as previously described [29].

### Primer extension assays

Six-well plates with confluent 293T cells were transiently transfected with a plasmid mixture containing 500 ng pcXN2 plasmid expressing EG/D1 PB2, PB1 and PA, 3 μg pcXN2 plasmid expressing EG/D1 NP and 500 ng pPol plasmid expressing EG/D1 segment 6 RNA. At 72 h post-transfection, cells were collected in Trizol reagent (Invitrogen) and RNA was purified according to the manufacturer’s protocol. Primer extension analysis was carried out using specific DIG-labeled primers for segment 6 vRNA and cRNA, with cell 5S ribosomal RNA (5S rRNA) as an internal control. Primer extension was carried out with SuperScript III reverse transcriptase (Invitrogen). After heating at 65°C for 5 min and quickly cooling on ice, transcription products were analyzed on 8% polyacrylamide gels containing 7 M urea in TBE buffer, transferred onto positively charged Nylon Membranes (Roche) and baked at 80°C for 4 h. For chemiluminescence detection, anti-DIG antibody linked to alkaline phosphatase, Fab and CDP-Star (Roche) were used according to the manufacturer’s instructions. The band intensities were quantified by ImageJ software and vRNA expression was calculated.

### Western blotting

293T cells were transfected with equal amounts of expression plasmids (250 ng PB2 and 500 ng PB1). At 16 h post-transfection, the cells were harvested with sample buffer and heated for 20 min at 100°C. The cell lysates were then separated by SDS-PAGE and transferred onto a polyvinylidene difluoride membrane (Millipore). Immunoblot analysis was performed using anti-influenza PB2 or PB1 antibody (GeneTex) and HRP-conjugated secondary antibody. The antibodies were visualized with Pierce Western Blotting Substrate Plus (Thermo Scientific) and exposed on Amersham Hyperfilm ECL (GE Healthcare). The band intensities were quantified by ImageJ software and total protein expression was calculated.

### Experimental infections in mice

Five-week-old female BALB/C mice (Japan SLC), under mixed anesthesia (medetomidine-butorphanol-midazolam), were inoculated intranasally with 75 μl samples of serial 10-fold dilutions of virus in PBS. The mice were observed daily for 14 d for weight loss and mortality. Mouse lungs were collected 3 and 6 d post-inoculation with 2.5×10^5^ FFU virus and virus titers were assayed as FFU in MDCK cells. For histopathology analysis, mouse lungs were collected 3 d post-inoculation with 2.5×10^5^ FFU virus, fixed in 4% buffered paraformaldehyde, embedded in paraffin, cut into 5 μm sections, stained with hematoxylin and eosin, and examined by light microscopy. Immunohistochemical staining for the H5 antigen was performed [30] on deparafinized sections using a monoclonal antibody (C43) specific for influenza A virus nucleoprotein and a Mouse on Mouse Peroxidase Kit (Vector) with diaminobenzidine as the chromogen and hematoxylin as the counterstain. Unrelated antibodies were used in place of the primary antibody as controls.

### Phylogenetic analysis

The sequences of 139 PB1 genes from influenza A virus subtype H5N1 strains isolated in Egypt from 2006–2011 were obtained from the GISAID database. Phylogenetic analysis was performed using MEGA4 software [50] for the neighbor-joining method, with the nucleotide sequences covering most of the PB1 gene. Estimates of the phylogenies were calculated by performing 1,000 bootstrap replicates.

### Homology modeling of EG/D1 mutants

The crystal structure of the heterotrimeric polymerase complex of influenza virus A/little yellow-shouldered bat/Guatemala/060/2010 (H17N10) bound to the vRNA promoter (Protein Data Bank ID code 4WSB) [31] was used as a template for homology modeling of EG/D1 and the indicated EG/D1 variants by the Molecular Operating Environment (MOE, http://www.chemcom.com). Residues 195–198 are missing in 4WSB and, therefore, the structure was built by MOE structure preparation tools (the parameter was default). Consensus vRNA promoter sequences (PDBID code 4WSB) were fit into the mutant models. After homology modeling, molecular optimization was carried out between the polymerase complexes and the vRNA promoter, and the interaction energy (IntE) and distance between each polymerase residue and the vRNA promoter residues in the complexes were calculated. The plausibility of this model was supported by the observation that the configuration of the EG/D1 structure modeled from the influenza A virus crystal structure (Protein Data Bank ID code 4WSB) corresponded to the polymerase complex crystal structure of influenza B virus (Protein Data Bank ID code 4WSA) including the complete PB1 β-ribbon [34], within a root mean square deviation (RMSD) of 2.055Å.

### Statistical analysis

Statistical analysis was carried out using GraphPad Prism Version 6 software (GraphPad Software Inc.).

## Supporting Information

S1 Fig Schematic diagram of the influenza virus minigenome assay. (A) In the vRNA-oriented minigenome assay, human polymerase I transcribes vRNA-like luciferase RNA, which is subsequently transcribed by the influenza virus polymerase complex and nucleoprotein. Thus, reporter mRNA can be transcribed directly from the vRNA-like molecule. (B) In the cRNA-oriented minigenome assay, human polymerase I transcribes cRNA-like luciferase RNA, which must be replicated to a vRNA-like molecule before reporter mRNA transcription. Thus, reporter gene expression is dependent on influenza virus polymerase-mediated replication in the cRNA-oriented minigenome assay. Thick arrows indicate RNA synthesis steps that should be efficient in each minigenome assay system.
(PDF)

S2 Fig Effect of the PB2-E627K mutation on clade 2.2.1 virus polymerase activity in human cells. 293T cells were transfected with plasmids expressing EG/D1 PB1, PA, NP, and EG/D1 PB2 (wt) or the PB2-K627E mutant, and with a human polymerase I-driven plasmid expressing a vRNA-luciferase reporter gene and a *Renilla* luciferase-expressing plasmid as an internal control. For comparison, cells were also transfected with plasmids expressing WSN (H1N1) PB2, PB1, PA and NP, and with the other plasmids listed above. After 48 h at 37°C, luciferase activities were measured and normalized to the internal *Renilla* luciferase activity. Each data point is the mean ± SD of three independent experiments. The asterisks indicate a *P* value <0.01 (ANOVA with Tukey’s multiple comparison test).
(PDF)

S3 Fig Effect of inter-segment combinations of mutations on polymerase activity. 293T and QT-6 cells were transfected with plasmids expressing an inter-segment combination of mutations in EG/D1 PB2, PB1, PA and NP as indicated, a human or chicken polymerase I-driven plasmid expressing a vRNA-oriented or cRNA-oriented luciferase reporter gene, and a *Renilla* luciferase-expressing plasmid as an internal control. Luciferase activities were assayed at 48 h post-transfection and normalized to the internal *Renilla* luciferase activity. The data were expressed relative to the results for EG/D1 (wt) polymerase. (A) Comparison of luciferase activity at 37 and 33°C in vRNA-oriented minigenome assays in 293T cells. (B) Comparison of luciferase activity at 37 and 33°C in cRNA-oriented minigenome assays in 293T cells. (C) Comparison of polymerase activity in 293T and QT-6 cells in vRNA-oriented minigenome assays at 37°C. (D) Comparison of polymerase activity in 293T cells and QT-6 cells in cRNA-oriented minigenome assays at 37°C. Each data point is the mean ± SD of three independent experiments. Bars with light and dark colors indicate single/intra-segment multiple mutations and inter-segment combinations of mutations, respectively.
(PDF)

S4 Fig Progeny vRNA productions by EG/D1 variants with polymerase mutations in primary human airway cells. SAE cells were infected with EG/D1 (wt) or a virus strain with the indicated polymerase mutation(s) at an MOI of 0.03 and incubated at 37°C (A) or 33°C (B). The culture supernatants were harvested at the indicated times post-infection and assayed by quantitative real-time RT-PCR to determine the amount of progeny vRNA. Each data point is the mean ± SD of the log10 number of vRNA copies/ml from three separate experiments. Single and double asterisks indicate a *P* value <0.05 and <0.01, respectively, when compared with the EG/D1 (wt) virus titer (Student’s *t* test).
(PDF)

S5 Fig Progeny vRNA productions by EG/D1 variants with polymerase mutations in avian cells. CEFs were infected with EG/D1 (wt) or a virus strain with the indicated polymerase mutation(s) at an MOI of 0.005 and incubated at 37°C. The culture supernatants were harvested at the indicated times post-infection and assayed by quantitative real-time RT-PCR to determine the amount of progeny virus RNA. Each data point is the mean ± SD of the log10 number of viral RNA copies/ml from three separate experiments.
(PDF)

S6 Fig Determination of vRNA and cRNA levels by primer extension assays after reconstitution of EG/D1 polymerase and NP with the indicated mutations in 293T cells. Primers specific for virus segment 6 were used in this assay. 5S rRNA levels served as an internal loading control. After quantitation of the band intensities using ImageJ software, the amount of expression for each vRNA (upper panel) was calculated relative to that for EG/D1 (wt). The asterisks indicate a *P* value <0.01 (ANOVA with Tukey’s multiple comparison test). Representative results of primer extension assays for EG/D1 and the indicated mutants are shown.
(PDF)

S7 Fig Expression of wild-type and mutant polymerase proteins in 293T cells. 293T cells were transfected with mutant PB2 or PB1 expression plasmids. At 16 h post-transfection, cells were harvested and protein expression levels were analyzed by Western blotting using anti-PB1 or PB2 antibodies. After quantitation of the band intensities using ImageJ software, the amount of expression for each PB1 (A, upper panel) or PB2 (B, upper panel) was calculated relative to that for EG/D1 (wt). Representative results of western blots for EG/D1 and the indicated PB1 mutants (A, lower panel) and PB2 mutants (B, lower panel) are shown. NS indicates statistically not significant (ANOVA).
(PDF)

S8 Fig Body weight of mice infected with EG/D1 strains with polymerase mutations. Five-week-old BALB/c mice (5 mice per group) were inoculated intranasally with serial 10-fold dilutions of the indicated viruses. The body weight of infected mice was monitored for 14 d post-infection. The mean ± SD of the percent body weight change for each group of mice is shown. The numbers in the graphs show the numbers of surviving animals.
(PDF)

S9 Fig Phylogenetic tree of the PB1 gene of clade 2.2.1 H5N1 viruses. This phylogenetic tree was reconstructed from sequence data for the PB1 genes of the 139 H5N1 clade 2.2.1 viruses available in the GISAID database. Subgroups of clade 2.2.1 viruses and the amino acid mutations that are conserved in each of the branches or specific to human virus strains are shown on the right. Green is used to highlight human virus strains.
(PDF)

S10 Fig Effect of mutations on structural changes in the polymerase complex. (A) A plausible structural model of the EG/D1 PB1 β-ribbon and β-hairpin with the vRNA promoter. The locations of vRNA contact sites and the mutations identified in this study are shown in blue and red, respectively. Other structures are omitted for clarity. (B−D) Mutant polymerase structures with the mutations identified in this study superimposed onto EG/D1 (wt) PB1, showing the flexibility of the long β-ribbon. (B) Close-up of residue 198 in the EG/D1 PB1 β-ribbon with vRNA, with the K198R mutation superimposed. (C) Close-up of vRNA contact residue 188 in the EG/D1 PB1 β-ribbon with vRNA (wt; green), with the mutant polymerase structures carrying K198R/D175N (red) and K198R/K214R (blue) superimposed. (D) Close-up of vRNA contact residue 203 in the EG/D1 PB1 β-ribbon with vRNA (wt, green), with the mutant polymerase structures carrying T182I/K214R (pink) and K198R/D175N (cyan) superimposed. (E) Close-up of residue 384 in the EG/D1 PB1 β-hairpin with vRNA, with the L384S mutation superimposed. Potential interactions between the vRNA contact residues and neighboring vRNA are represented by broken lines and distances (Å) are indicated with the same colors as the mutant polymerase structures.
(PDF)

S11 Fig Phylogenetic tree of the PB2 gene of clade 2.2.1 H5N1 viruses. This phylogenetic tree was reconstructed from sequence data for the PB2 genes of the 152 H5N1 clade 2.2.1 viruses available in the GISAID database. Subgroups of clade 2.2.1 viruses and the amino acid mutations that are conserved in each of the branches or specific to human virus strains are shown on the right. Green is used to highlight human virus strains.
(PDF)

S12 Fig Phylogenetic tree of the PA gene of clade 2.2.1 H5N1 viruses. This phylogenetic tree was reconstructed from sequence data for the PA genes of the 139 H5N1 clade 2.2.1 viruses available in the GISAID database. Subgroups of clade 2.2.1 viruses and the amino acid mutations that are conserved in each of the branches or specific to human virus strains are shown on the right. Green is used to highlight human virus strains.
(PDF)

S13 Fig Phylogenetic tree of the NP gene of clade 2.2.1 H5N1 viruses. This phylogenetic tree was reconstructed from sequence data for the NP genes of the 142 H5N1 clade 2.2.1 viruses available in the GISAID database. Subgroups of clade 2.2.1 viruses and the amino acid mutations that are conserved in each of the branches or specific to human virus strains are shown on the right. Green is used to highlight human virus strains.
(PDF)

S1 Table Identified mutations in the polymerase sequences of the 61 H5N1 viruses isolated from Egyptian patients by database search. (PDF)

S2 Table Prevalence the of clade 2.2.1 virus polymerase mutations identified in the database search in this study. (PDF)

S3 Table Summary of the results from various assays in this study. (PDF)

S4 Table Interaction energy between the vRNA promoter and vRNA contact residues in EG/D1 (wt) PB1 and PB1 mutants. (PDF)
