# Supplementary figures and images for: Novel Polymerase Gene Mutations for Human Adaptation in Clinical Isolates of Avian H5N1 Influenza Viruses
Source: PLoS Pathog. 2016 Apr 20;12(4):e1005583. doi: 10.1371/journal.ppat.1005583 (PMC4838241; doi:10.1371/journal.ppat.1005583)

**A**

**vRNA-oriented minigenome assay**

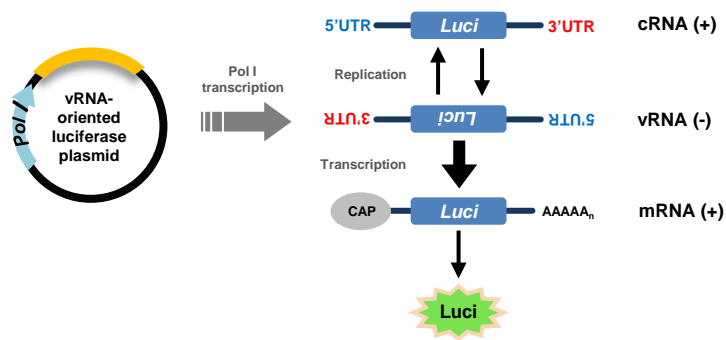

**B**

**cRNA-oriented minigenome assay**

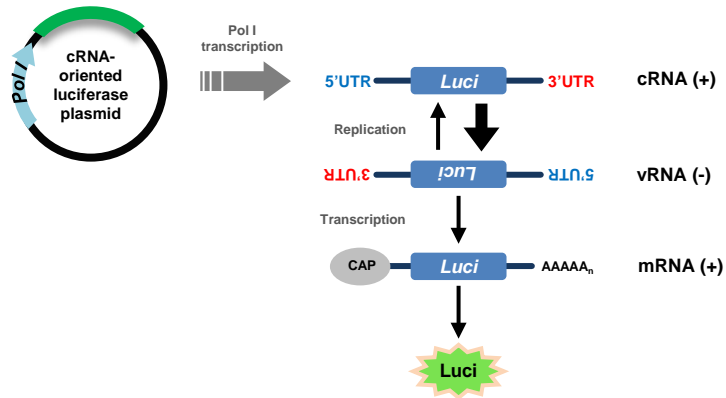

Supplement: S1 Fig — (A) In the vRNA-oriented minigenome assay, human polymerase I transcribes vRNA-like luciferase RNA, which is subsequently transcribed by the influenza virus polymerase complex and nucleoprotein. Thus, reporter mRNA can be transcribed directly from the vRNA-like molecule. (B) In the cRNA-oriented minigenome assay, human polymerase I transcribes cRNA-like luciferase RNA, which must be replicated to a vRNA-like molecule before reporter mRNA transcription. Thus, reporter gene expression is dependent on influenza virus polymerase-mediated replication in the cRNA-oriented minigenome assay. Thick arrows indicate RNA synthesis steps that should be efficient in each minigenome assay system. (PDF) [file ppat.1005583.s001.pdf]

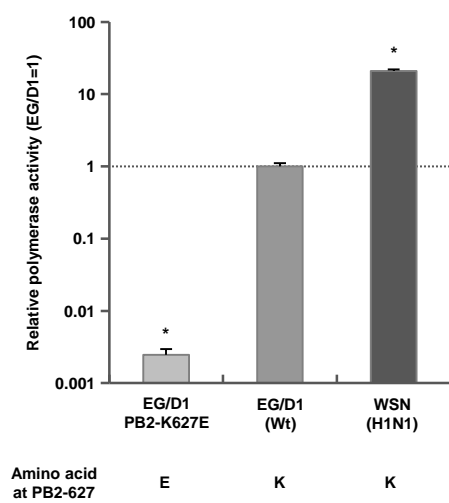

Supplement: S2 Fig — 293T cells were transfected with plasmids expressing EG/D1 PB1, PA, NP, and EG/D1 PB2 (wt) or the PB2-K627E mutant, and with a human polymerase I-driven plasmid expressing a vRNA-luciferase reporter gene and a Renilla luciferase-expressing plasmid as an internal control. For comparison, cells were also transfected with plasmids expressing WSN (H1N1) PB2, PB1, PA and NP, and with the other plasmids listed above. After 48 h at 37°C, luciferase activities were measured and normalized to the internal Renilla luciferase activity. Each data point is the mean ± SD of three independent experiments. The asterisks indicate a P value <0.01 (ANOVA with Tukey’s multiple comparison test). (PDF) [file ppat.1005583.s002.pdf]

**A**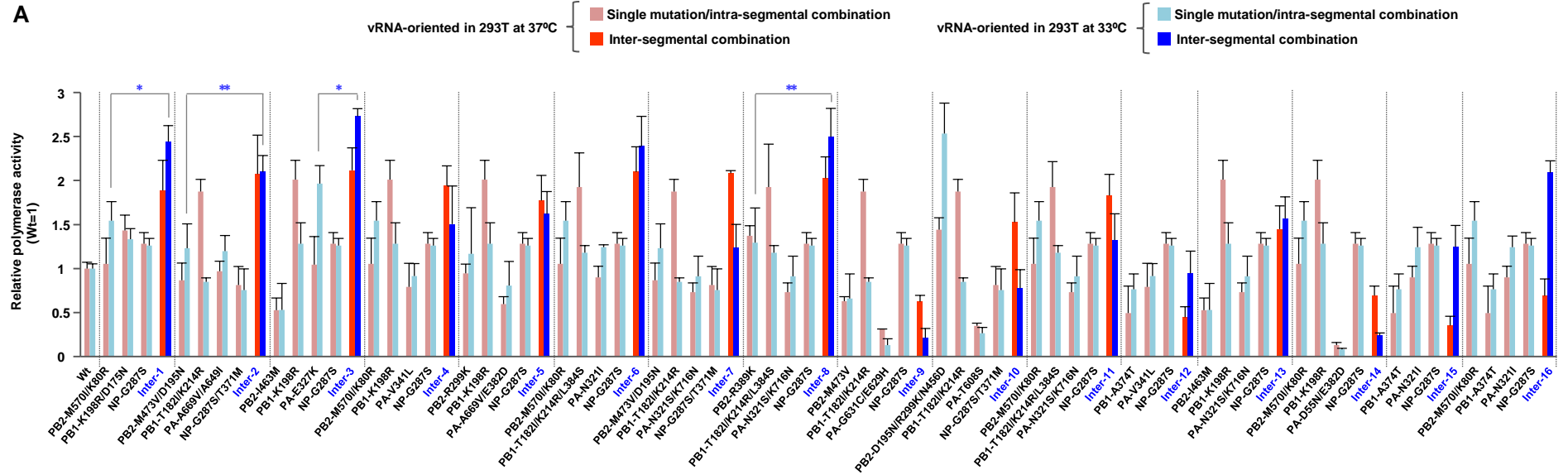**B**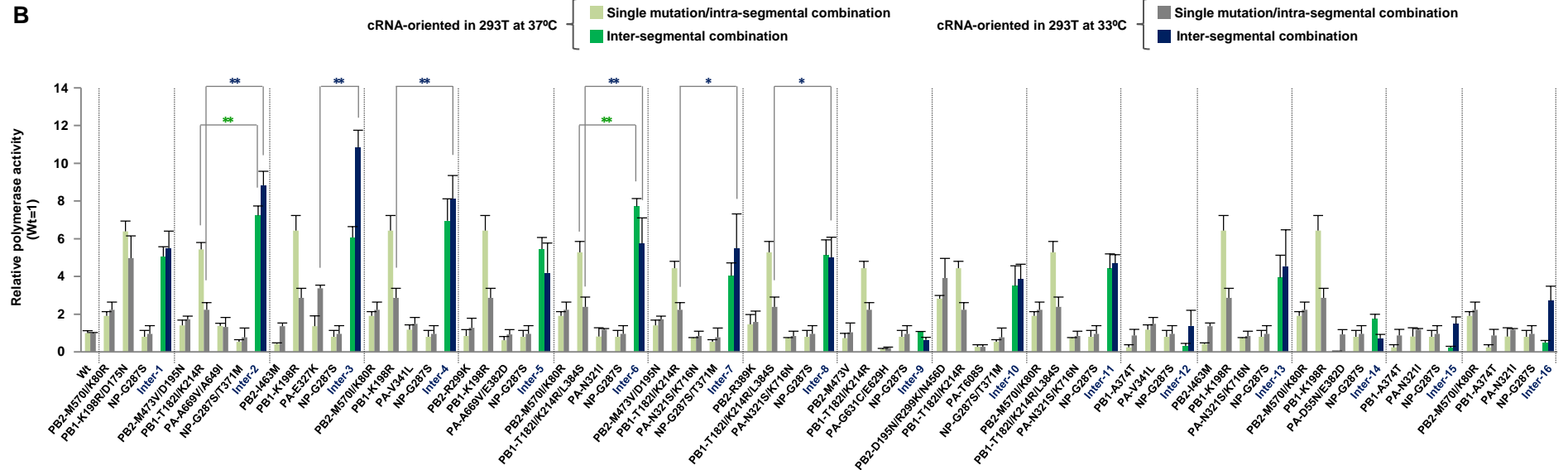

C

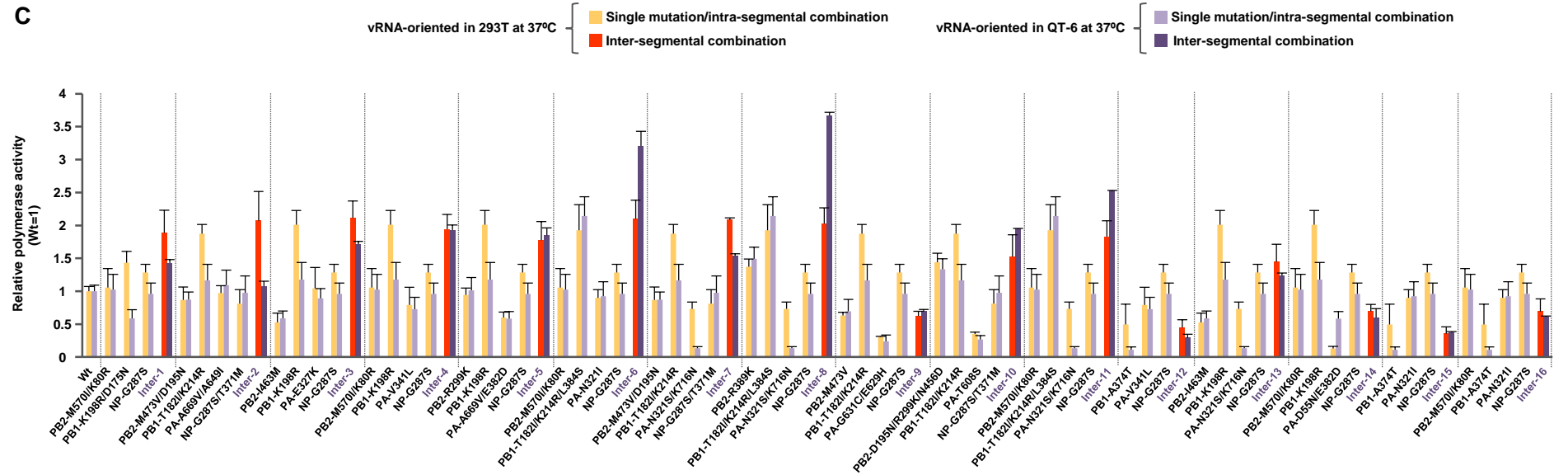

D

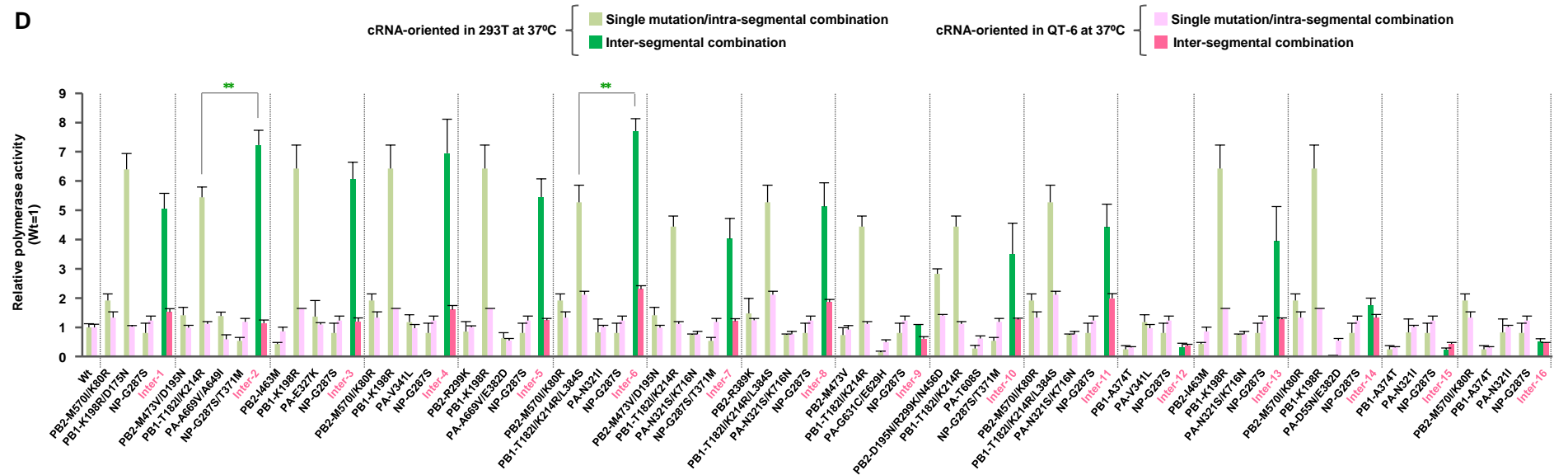

Supplement: S3 Fig — 293T and QT-6 cells were transfected with plasmids expressing an inter-segment combination of mutations in EG/D1 PB2, PB1, PA and NP as indicated, a human or chicken polymerase I-driven plasmid expressing a vRNA-oriented or cRNA-oriented luciferase reporter gene, and a Renilla luciferase-expressing plasmid as an internal control. Luciferase activities were assayed at 48 h post-transfection and normalized to the internal Renilla luciferase activity. The data were expressed relative to the results for EG/D1 (wt) polymerase. (A) Comparison of luciferase activity at 37 and 33°C in vRNA-oriented minigenome assays in 293T cells. (B) Comparison of luciferase activity at 37 and 33°C in cRNA-oriented minigenome assays in 293T cells. (C) Comparison of polymerase activity in 293T and QT-6 cells in vRNA-oriented minigenome assays at 37°C. (D) Comparison of polymerase activity in 293T cells and QT-6 cells in cRNA-oriented minigenome assays at 37°C. Each data point is the mean ± SD of three independent experiments. Bars with light and dark colors indicate single/intra-segment multiple mutations and inter-segment combinations of mutations, respectively. (PDF) [file ppat.1005583.s003.pdf]

A

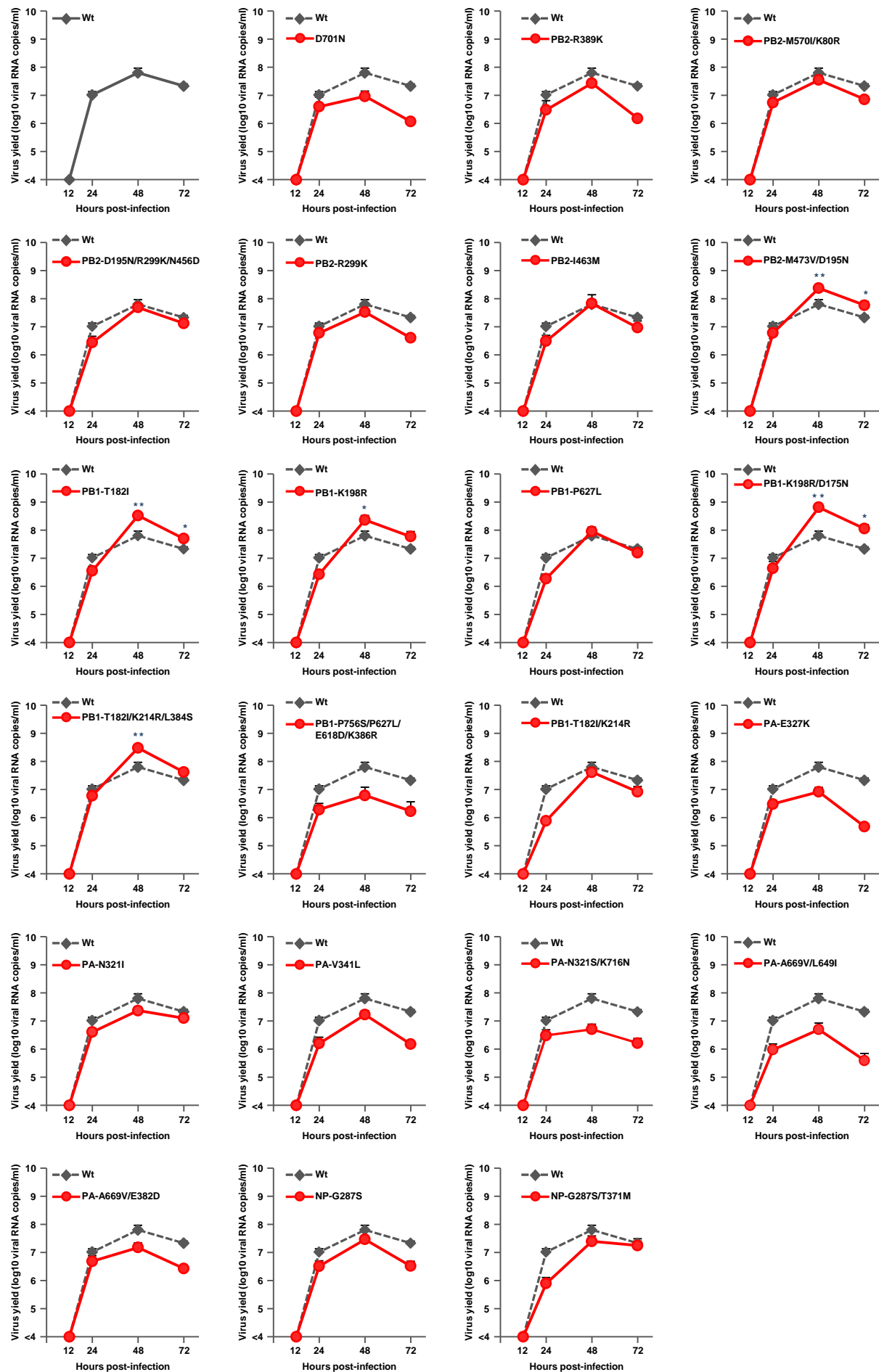

A (Continued)

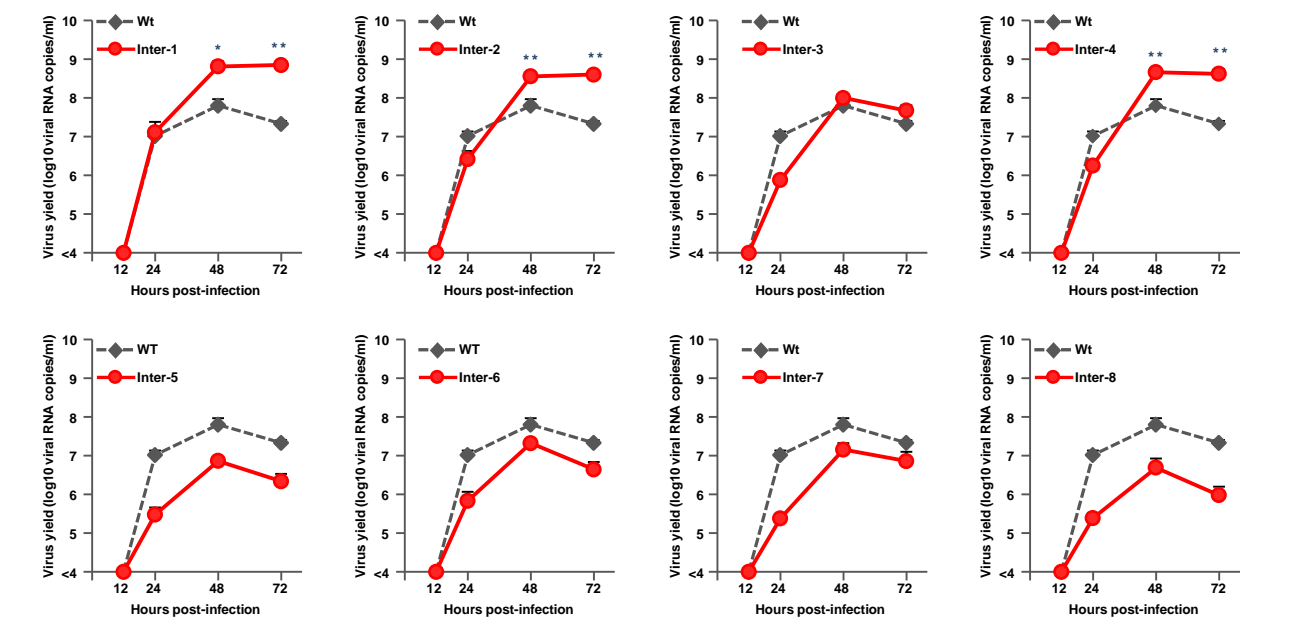

**B**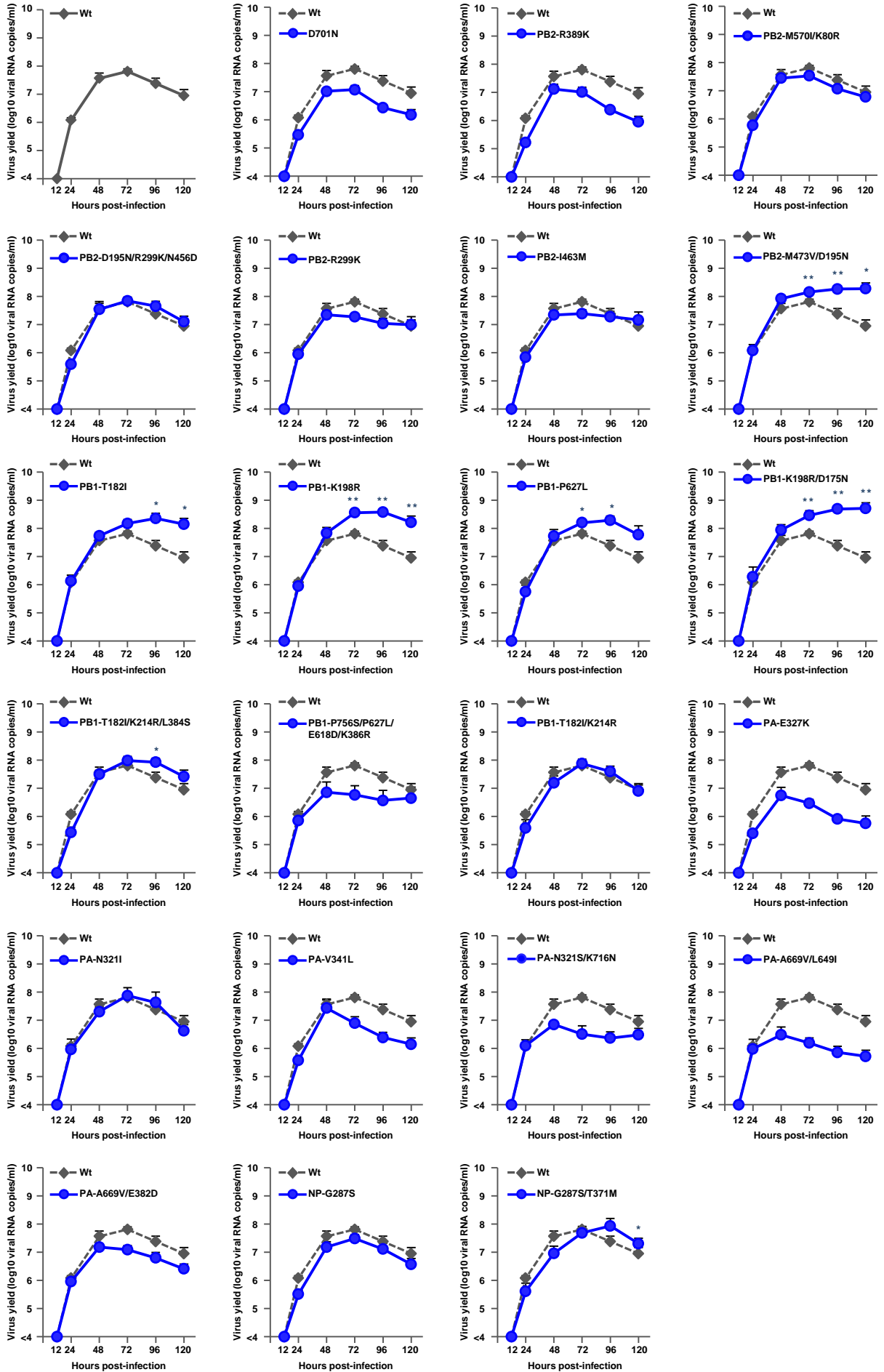

B (Continued)

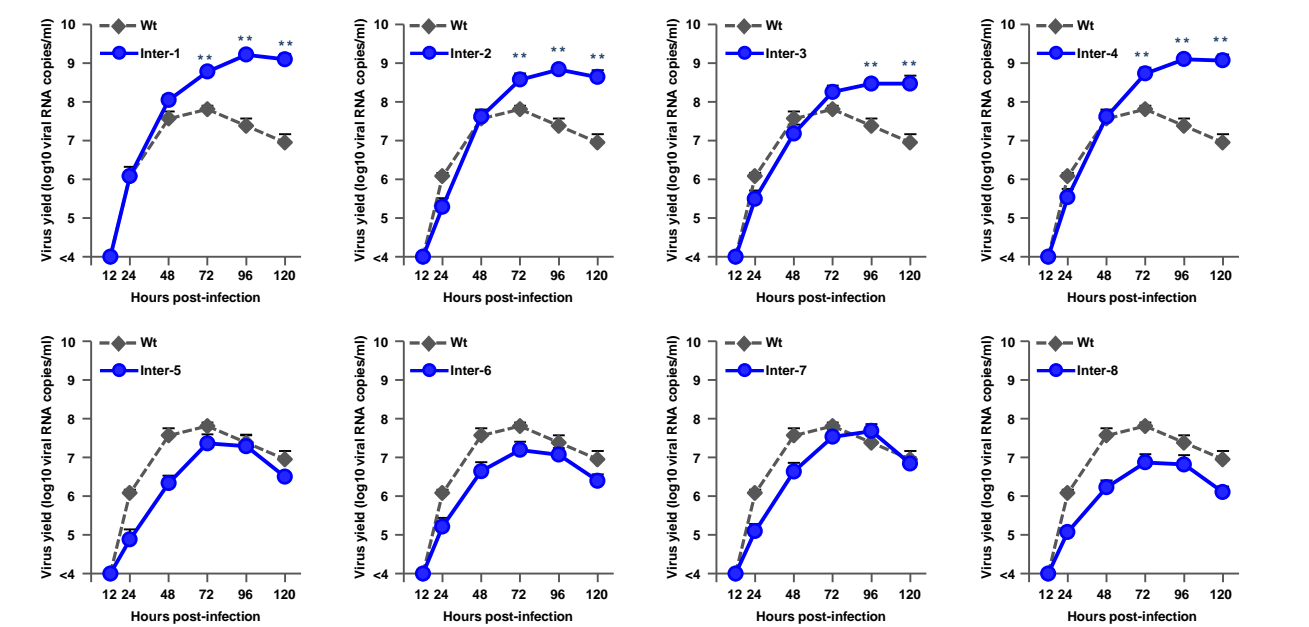

Supplement: S4 Fig — SAE cells were infected with EG/D1 (wt) or a virus strain with the indicated polymerase mutation(s) at an MOI of 0.03 and incubated at 37°C (A) or 33°C (B). The culture supernatants were harvested at the indicated times post-infection and assayed by quantitative real-time RT-PCR to determine the amount of progeny vRNA. Each data point is the mean ± SD of the log10 number of vRNA copies/ml from three separate experiments. Single and double asterisks indicate a P value <0.05 and <0.01, respectively, when compared with the EG/D1 (wt) virus titer (Student’s t test). (PDF) [file ppat.1005583.s004.pdf]

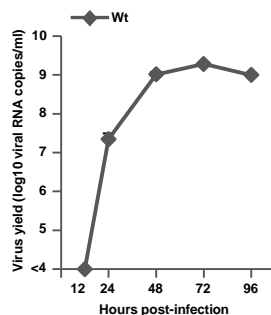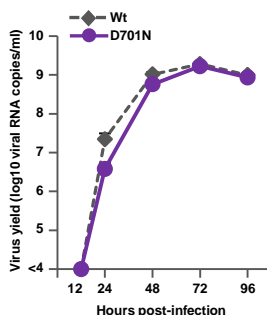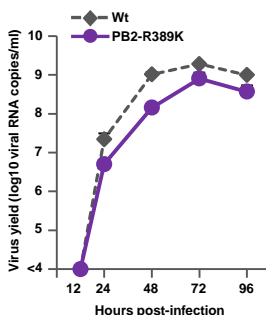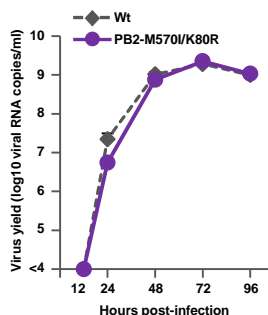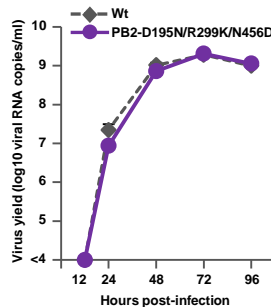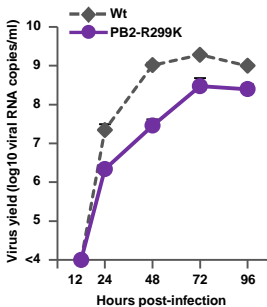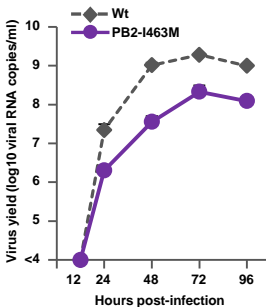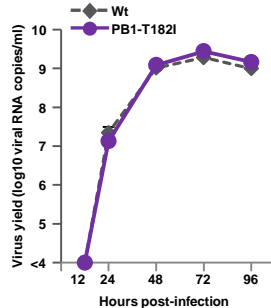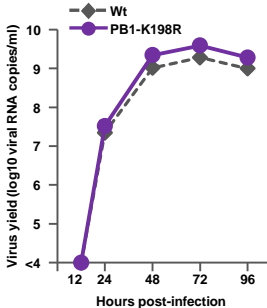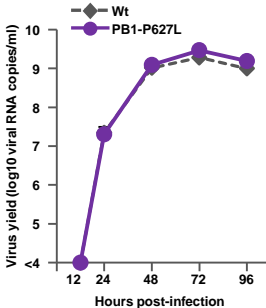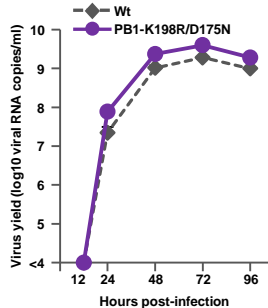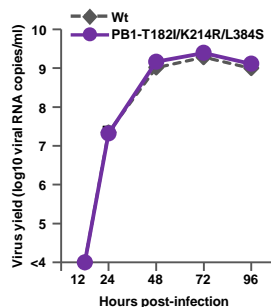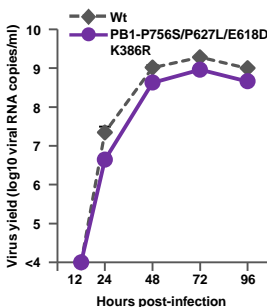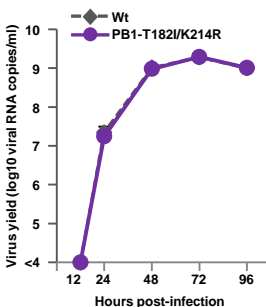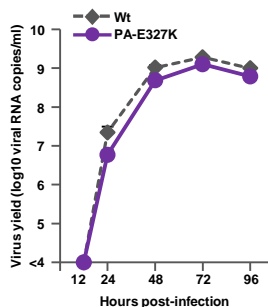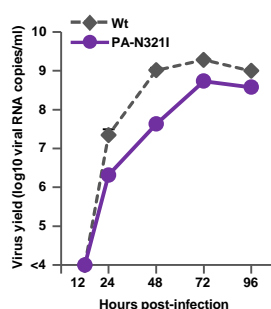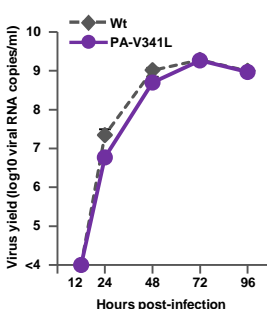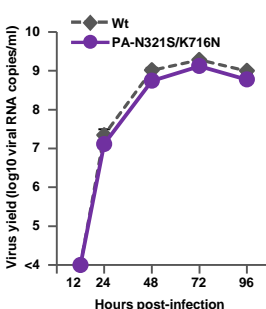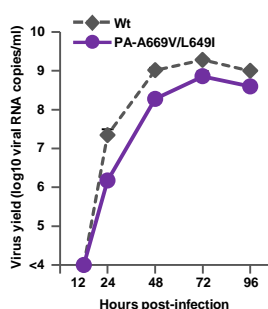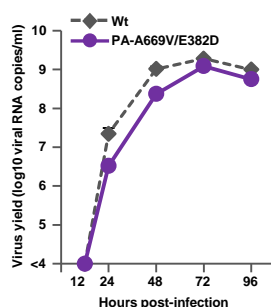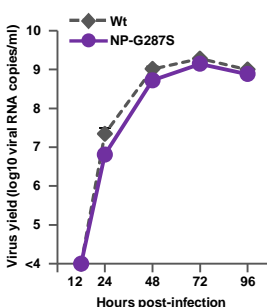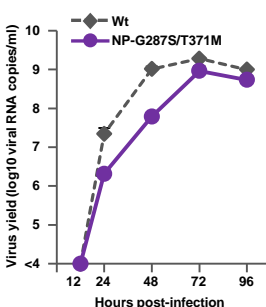

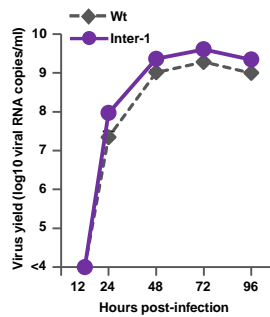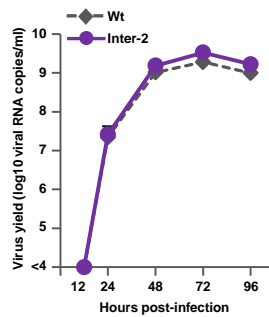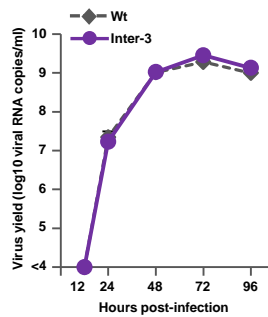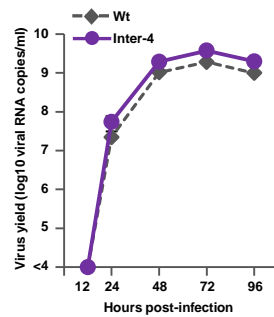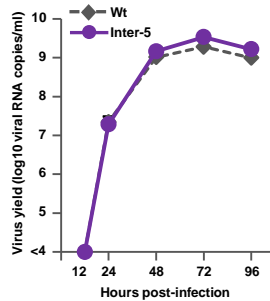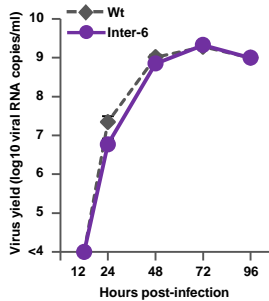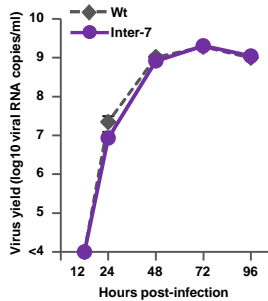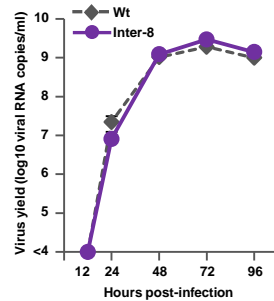

Supplement: S5 Fig — CEFs were infected with EG/D1 (wt) or a virus strain with the indicated polymerase mutation(s) at an MOI of 0.005 and incubated at 37°C. The culture supernatants were harvested at the indicated times post-infection and assayed by quantitative real-time RT-PCR to determine the amount of progeny virus RNA. Each data point is the mean ± SD of the log10 number of viral RNA copies/ml from three separate experiments. (PDF) [file ppat.1005583.s005.pdf]

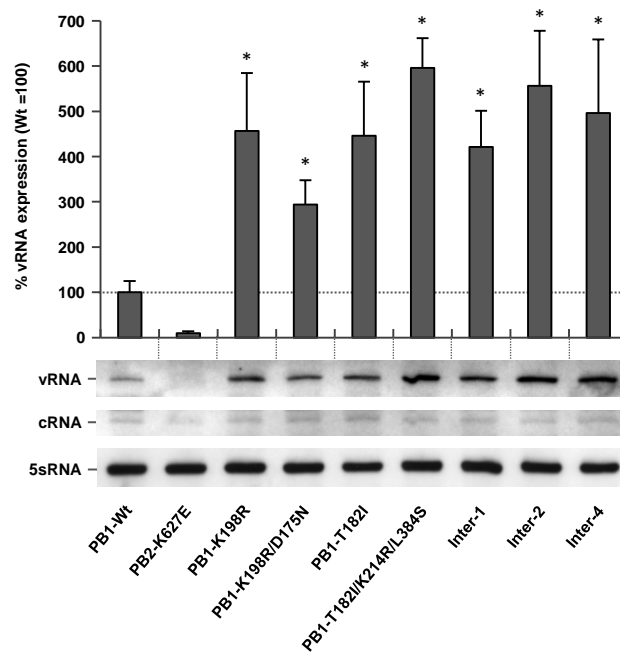

Supplement: S6 Fig — Primers specific for virus segment 6 were used in this assay. 5S rRNA levels served as an internal loading control. After quantitation of the band intensities using ImageJ software, the amount of expression for each vRNA (upper panel) was calculated relative to that for EG/D1 (wt). The asterisks indicate a P value <0.01 (ANOVA with Tukey’s multiple comparison test). Representative results of primer extension assays for EG/D1 and the indicated mutants are shown. (PDF) [file ppat.1005583.s006.pdf]

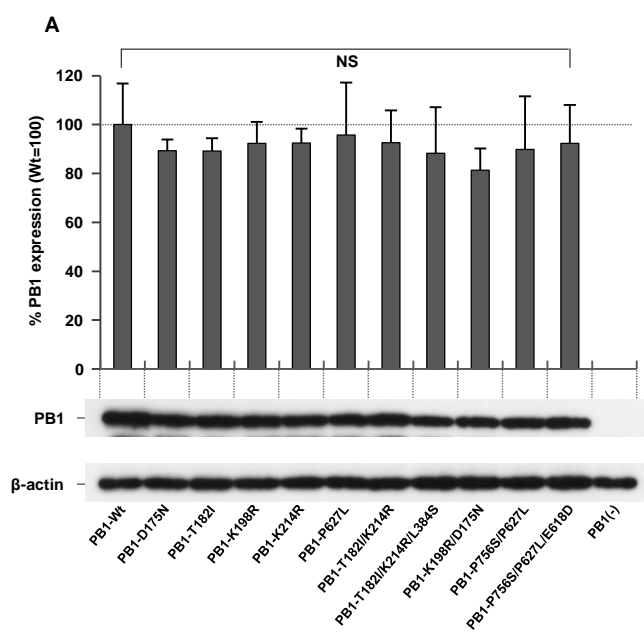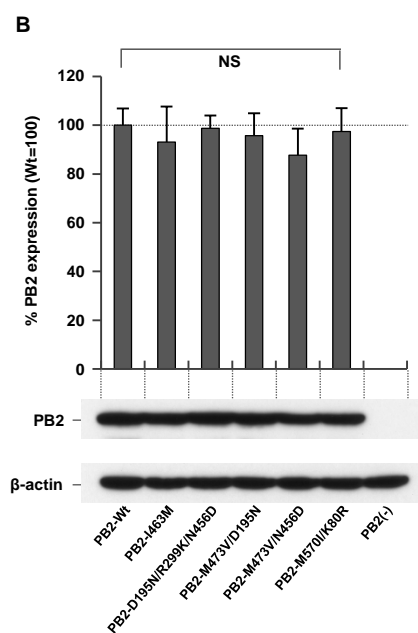

Supplement: S7 Fig — 293T cells were transfected with mutant PB2 or PB1 expression plasmids. At 16 h post-transfection, cells were harvested and protein expression levels were analyzed by Western blotting using anti-PB1 or PB2 antibodies. After quantitation of the band intensities using ImageJ software, the amount of expression for each PB1 (A, upper panel) or PB2 (B, upper panel) was calculated relative to that for EG/D1 (wt). Representative results of western blots for EG/D1 and the indicated PB1 mutants (A, lower panel) and PB2 mutants (B, lower panel) are shown. NS indicates statistically not significant (ANOVA). (PDF) [file ppat.1005583.s007.pdf]

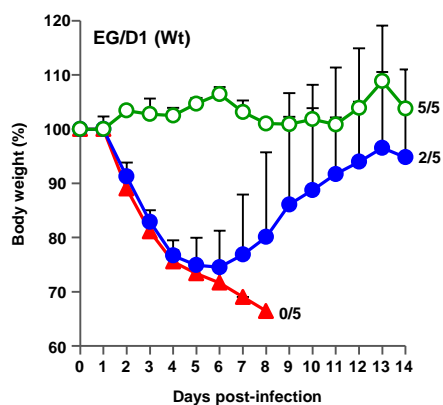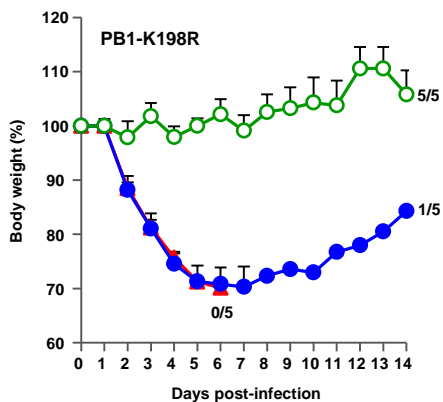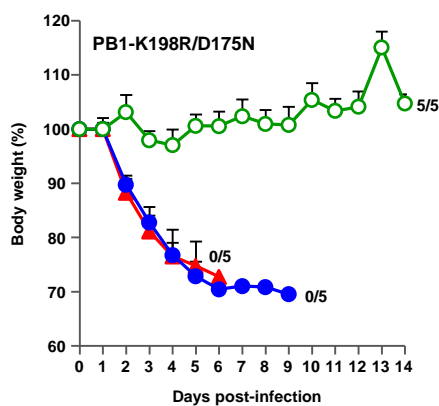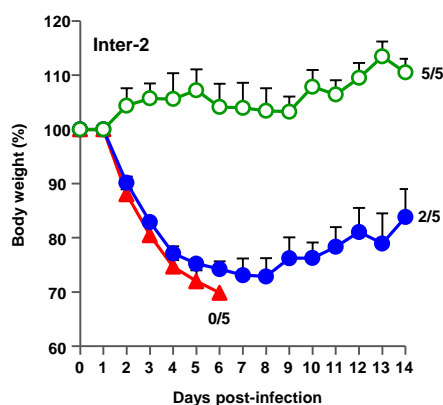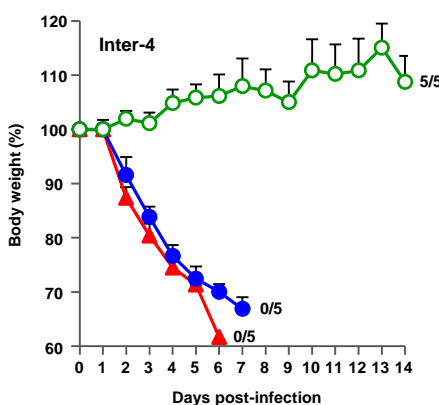

Supplement: S8 Fig — Five-week-old BALB/c mice (5 mice per group) were inoculated intranasally with serial 10-fold dilutions of the indicated viruses. The body weight of infected mice was monitored for 14 d post-infection. The mean ± SD of the percent body weight change for each group of mice is shown. The numbers in the graphs show the numbers of surviving animals. (PDF) [file ppat.1005583.s008.pdf]

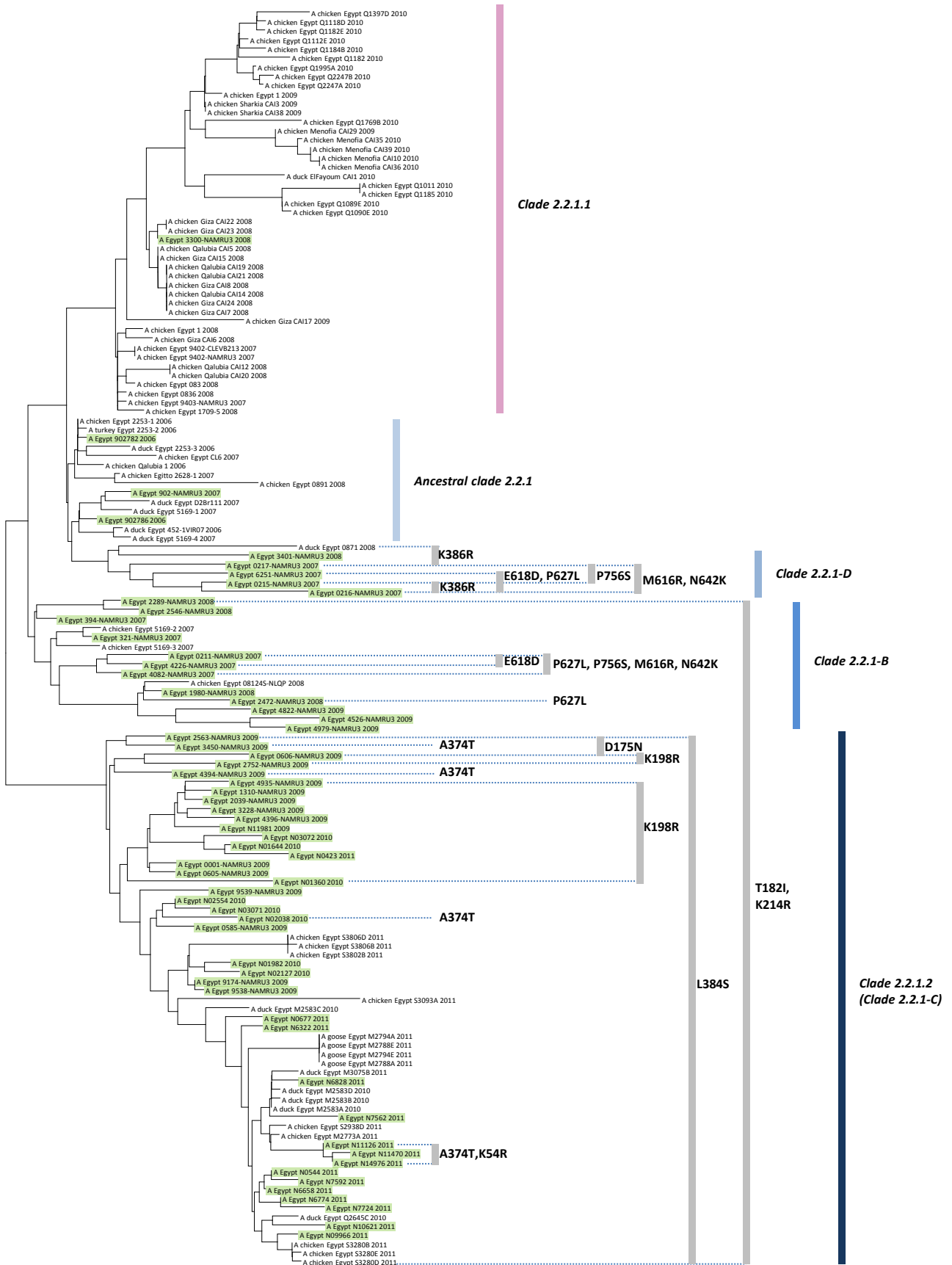

Supplement: S9 Fig — This phylogenetic tree was reconstructed from sequence data for the PB1 genes of the 139 H5N1 clade 2.2.1 viruses available in the GISAID database. Subgroups of clade 2.2.1 viruses and the amino acid mutations that are conserved in each of the branches or specific to human virus strains are shown on the right. Green is used to highlight human virus strains. (PDF) [file ppat.1005583.s009.pdf]

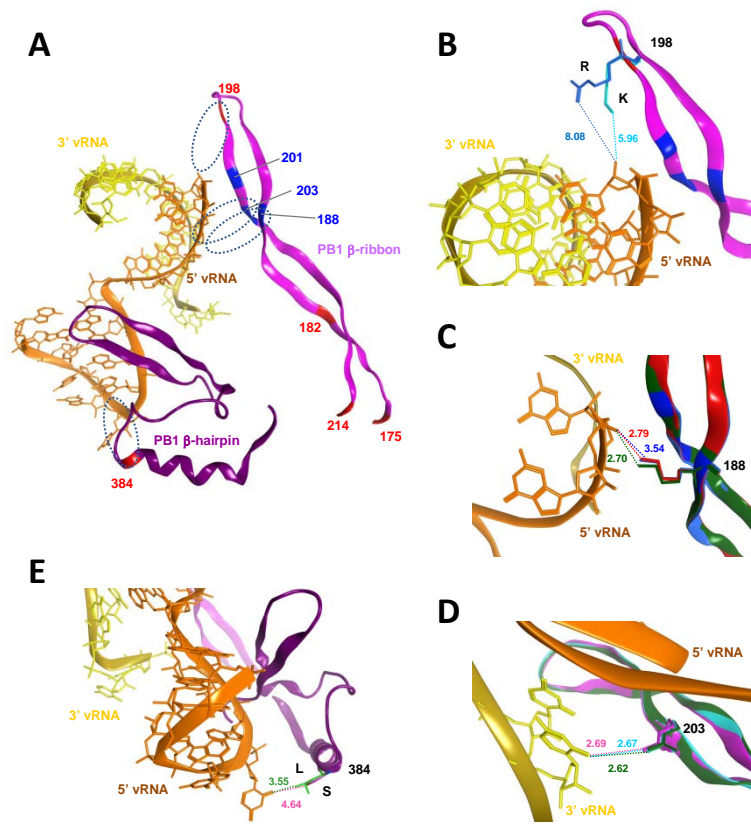

Supplement: S10 Fig — (A) A plausible structural model of the EG/D1 PB1 β-ribbon and β-hairpin with the vRNA promoter. The locations of vRNA contact sites and the mutations identified in this study are shown in blue and red, respectively. Other structures are omitted for clarity. (B−D) Mutant polymerase structures with the mutations identified in this study superimposed onto EG/D1 (wt) PB1, showing the flexibility of the long β-ribbon. (B) Close-up of residue 198 in the EG/D1 PB1 β-ribbon with vRNA, with the K198R mutation superimposed. (C) Close-up of vRNA contact residue 188 in the EG/D1 PB1 β-ribbon with vRNA (wt; green), with the mutant polymerase structures carrying K198R/D175N (red) and K198R/K214R (blue) superimposed. (D) Close-up of vRNA contact residue 203 in the EG/D1 PB1 β-ribbon with vRNA (wt, green), with the mutant polymerase structures carrying T182I/K214R (pink) and K198R/D175N (cyan) superimposed. (E) Close-up of residue 384 in the EG/D1 PB1 β-hairpin with vRNA, with the L384S mutation superimposed. Potential interactions between the vRNA contact residues and neighboring vRNA are represented by broken lines and distances (Å) are indicated with the same colors as the mutant polymerase structures. (PDF) [file ppat.1005583.s010.pdf]

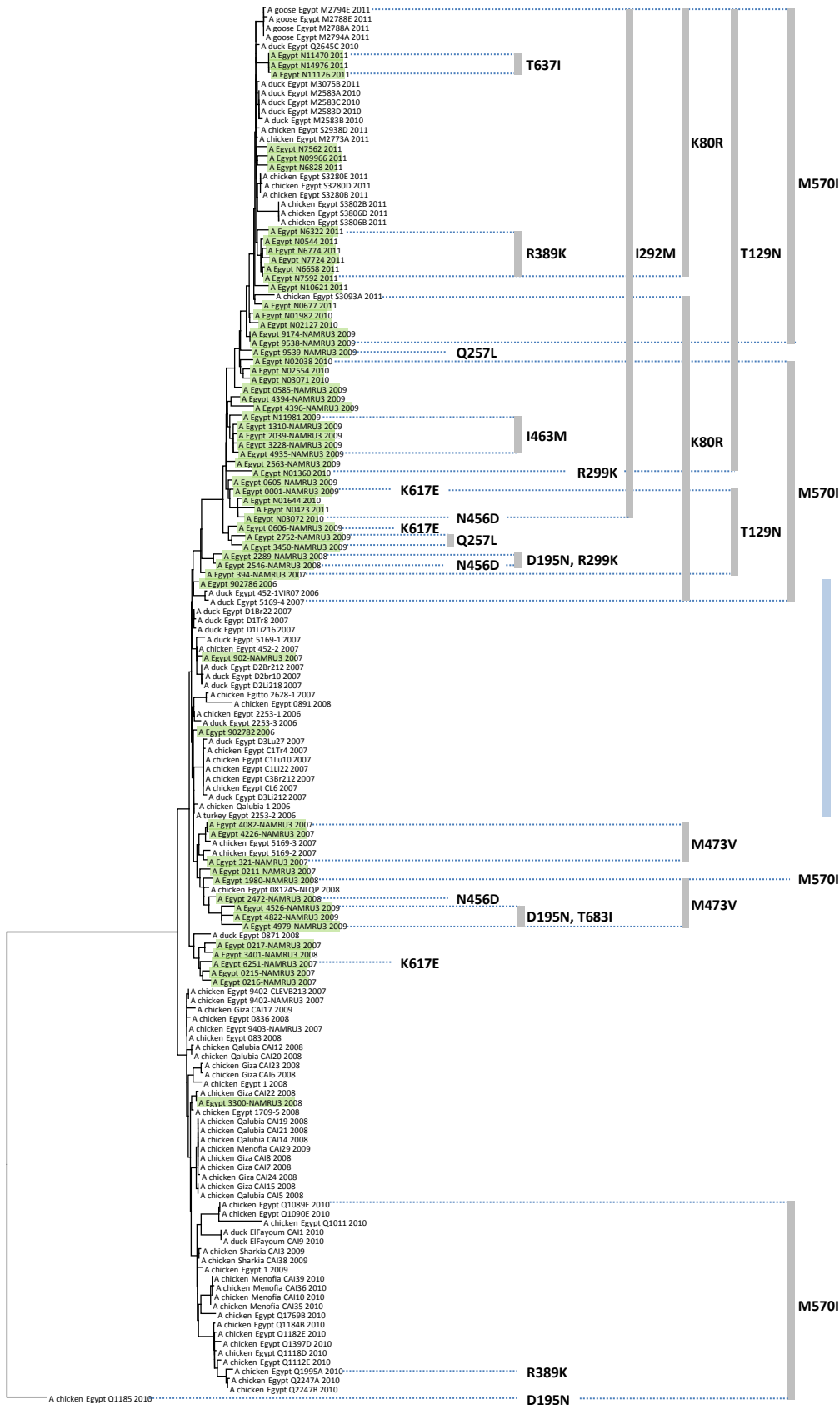

Clade 2.2.1.2  
(Clade 2.2.1-C)

Ancestral clade 2.2.1

Clade 2.2.1-B

Clade 2.2.1-D

Clade 2.2.1.1

Supplement: S11 Fig — This phylogenetic tree was reconstructed from sequence data for the PB2 genes of the 152 H5N1 clade 2.2.1 viruses available in the GISAID database. Subgroups of clade 2.2.1 viruses and the amino acid mutations that are conserved in each of the branches or specific to human virus strains are shown on the right. Green is used to highlight human virus strains. (PDF) [file ppat.1005583.s011.pdf]

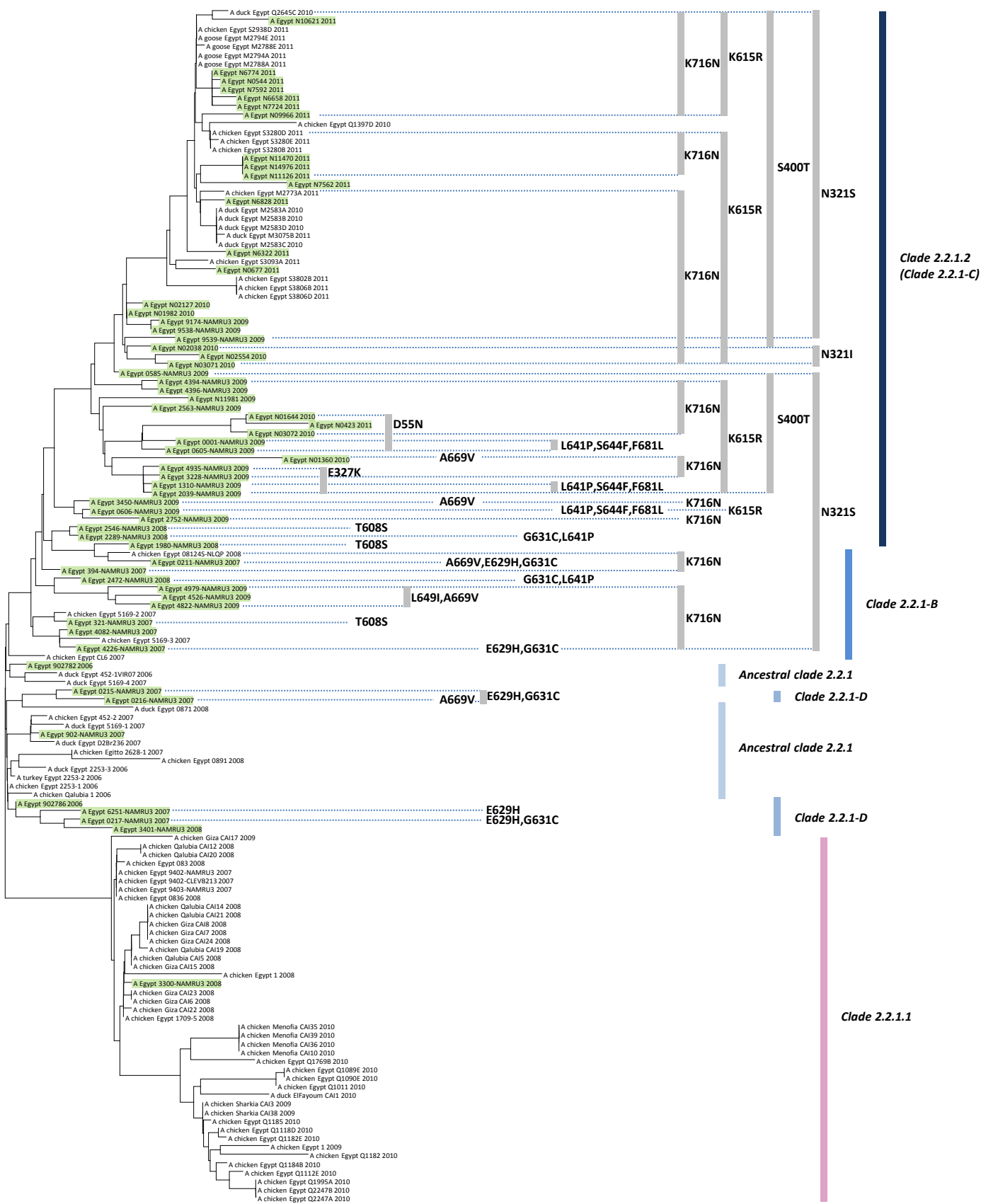

Supplement: S12 Fig — This phylogenetic tree was reconstructed from sequence data for the PA genes of the 139 H5N1 clade 2.2.1 viruses available in the GISAID database. Subgroups of clade 2.2.1 viruses and the amino acid mutations that are conserved in each of the branches or specific to human virus strains are shown on the right. Green is used to highlight human virus strains. (PDF) [file ppat.1005583.s012.pdf]

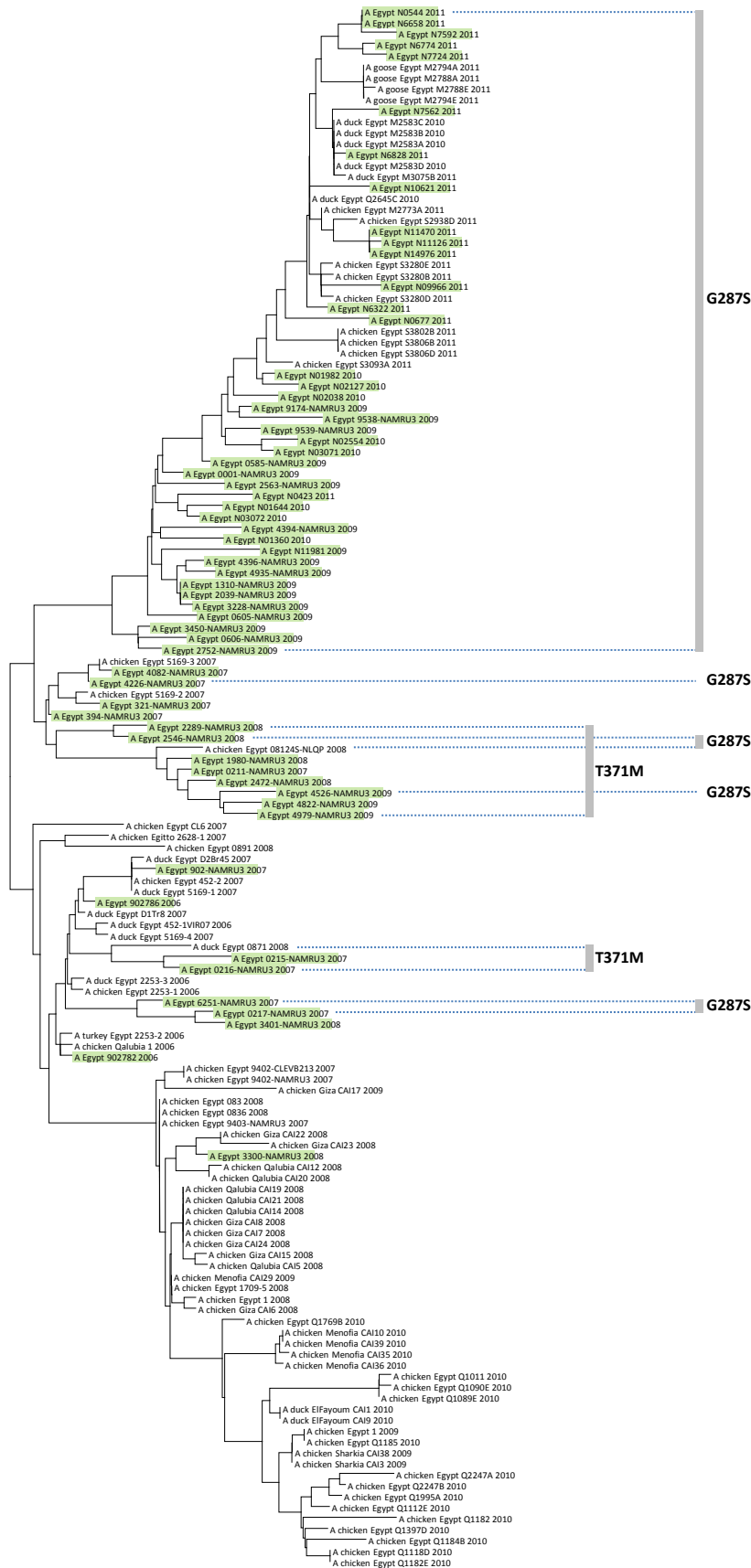

Clade 2.2.1.2  
(Clade 2.2.1-C)

Clade 2.2.1-B

Ancestral clade 2.2.1

Clade 2.2.1-D

Ancestral clade 2.2.1

Clade 2.2.1.1

Supplement: S13 Fig — This phylogenetic tree was reconstructed from sequence data for the NP genes of the 142 H5N1 clade 2.2.1 viruses available in the GISAID database. Subgroups of clade 2.2.1 viruses and the amino acid mutations that are conserved in each of the branches or specific to human virus strains are shown on the right. Green is used to highlight human virus strains. (PDF) [file ppat.1005583.s013.pdf]
