# Supplementary material for: Novel Polymerase Gene Mutations for Human Adaptation in Clinical Isolates of Avian H5N1 Influenza Viruses
Source: PLoS Pathog. 2016 Apr 20;12(4):e1005583. doi: 10.1371/journal.ppat.1005583 (PMC4838241; doi:10.1371/journal.ppat.1005583)
Supplement: S1 Table — (PDF) [file ppat.1005583.s014.pdf]

S1 Table. Identified mutations in the polymerase sequences of the 61 H5N1 viruses isolated from Egyptian patients by database search.

Clade	Virus Strain	Subtype	Passage history[†]	Host	Collection date	Host age	Host gender	Mutations in gene[*]			
								PB2	PB1	PA	NP
2.2.1	A/Egypt/902782/2006	H5N1		Human	2006	*U*[‡]	*U*[‡]				
2.2.1	A/Egypt/902786/2006	H5N1		Human	2006	*U*[‡]	*U*[‡]	M570I			
2.2.1	A/Egypt/394-NAMRU3/2007	H5N1	E1	Human	2007-02-05	17	Female	M570I/K80R/T129N	T182I/K214R	N321S	
2.2.1	A/Egypt/321-NAMRU3/2007	H5N1	E1	Human	2007-03-13	*U*[‡]	*U*[‡]	M473V	T182I/K214R	N321S/T608S	
2.2.1	A/Egypt/902-NAMRU3/2007	H5N1	E1	Human	2007-02-28	*U*[‡]	*U*[‡]				
2.2.1	A/Egypt/4226-NAMRU3/2007	H5N1	C1E1/E1	Human	2007-06-21	4	Female	M473V	T182I/K214R/M616R/P756S/P627L/E618D/N642K	N321S/E629H/G631C	G287S
2.2.1	A/Egypt/4082-NAMRU3/2007	H5N1	E2/E1	Human	2007-06-10	4	Female	M473V	T182I/K214R/M616R/P756S/P627L/N642K	N321S	
2.2.1	A/Egypt/6251-NAMRU3/2007	H5N1	C1/E2	Human	2007-07-21	25	Female	K617E	M616R/P756S/P627L/E618D/N642K	E629H	G287S
2.2.1	A/Egypt/0211-NAMRU3/2007	H5N1	E1/E1	Human	2007-12-24	25	Female		T182I/K214R/M616R/P756S/P627L/E618D/N642K	N321S/E629H/G631C/A669V	T371M
2.2.1	A/Egypt/0215-NAMRU3/2007	H5N1	E3/E1	Human	2007-12-26	22	Female		M616R/P756S/P627L/E618D/N642K/K386R	E629H/G631C	T371M
2.2.1	A/Egypt/0216-NAMRU3/2007	H5N1	E2/E1	Human	2007-12-29	36	Female		M616R/P627L/E618D/N642K/K386R	E629H/G631C/A669V/V341L	T371M
2.2.1	A/Egypt/0217-NAMRU3/2007	H5N1	E2/E1	Human	2007-12-29	30	Female		M616R/P756S/N642K/K386R	E629H/G631C	G287S
2.2.1	A/Egypt/1980-NAMRU3/2008	H5N1	E1/E1	Human	2008-02-26	4	Female	M570I/M473V	T182I/K214R	N321S/T608S/F681L	T371M
2.2.1	A/Egypt/2289-NAMRU3/2008	H5N1	E1/E1	Human	2008-03-02	25	Female	M570I/K80R/T129N/D195N/R299K	T182I/K214R	N321S/G631C/L641P	T371M
2.2.1	A/Egypt/2546-NAMRU3/2008	H5N1	C1/E1	Human	2008-03-08	8	Male	M570I/K80R/T129N/D195N/R299K/N456D	T182I/K214R	N321S/T608S	G287S/T371M
2.2.1	A/Egypt/2472-NAMRU3/2008	H5N1	C1/E1	Human	2008	19	Male	N456D/M473V	T182I/K214R	N321S/G631C/L641P	T371M
2.2.1	A/Egypt/3300-NAMRU3/2008	H5N1	C1/C1	Human	2008-04-11	30	Female				
2.2.1	A/Egypt/3401-NAMRU3/2008	H5N1	C1/C1	Human	2008-04-16	2	Male		K386R		
2.2.1	A/Egypt/0605-NAMRU3/2009	H5N1	C1E1/E1	Human	2009-02-03	2	Male	M570I/K80R/I292M	T182I/K214R/L384S/K198R	N321S/S400T/K615R/D55N/L641P/S644F/F681L	G287S
2.2.1	A/Egypt/0606-NAMRU3/2009	H5N1	E2/E1	Human	2009-02-07	1.5	Male	M570I/K80R/T129N/K617E	T182I/K214R/L384S/K198R/D175N	N321S/K615R/L641P/S644F/F681L	G287S
2.2.1	A/Egypt/1310-NAMRU3/2009	H5N1	E1/E1	Human	2009-02-28	2	Male	M570I/K80R/T129N/I292M/I463M	T182I/K214R/L384S/K198R	N321S/S400T/K615R/E327K/L641P/S644F/F681L	G287S
2.2.1	A/Egypt/2039-NAMRU3/2009	H5N1	C1/E1	Human	2009-03-03	2.5	Male	M570I/K80R/T129N/I292M/I463M	T182I/K214R/L384S/K198R	N321S/S400T/K615R/E327K/L641P/S644F/F681L	G287S
2.2.1	A/Egypt/0001-NAMRU3/2009	H5N1	C1/E1	Human	2009-01-01	1	Female	M570I/K80R/T129N/I292M/K617E	T182I/K214R/L384S/K198R	N321S/S400T/K615R/D55N/L641P/S644F/F681L	G287S
2.2.1	A/Egypt/0585-NAMRU3/2009	H5N1	C1/E1	Human	2009-01-23	2	Female	M570I/K80R/T129N/I292M	T182I/K214R/L384S	N321S/S400T	G287S
2.2.1	A/Egypt/9538-NAMRU3/2009	H5N1	E1/E1	Human	2009-05-18	*U*[‡]	*U*[‡]	M570I/K80R/T129N/I292M	T182I/K214R/L384S	N321S/S400T/K615R/K716N	G287S
2.2.1	A/Egypt/4526-NAMRU3/2009	H5N1	E1/E1	Human	2009-05-18	4	Male	D195N/M473V/T683I	T182I/K214R	N321S/K716N/L649I/A669V	G287S/T371M
2.2.1	A/Egypt/9539-NAMRU3/2009	H5N1	E1/E1	Human	2009-09-16	13	Male	K80R/T129N/I292M/Q257L	T182I/K214R/L384S	N321S/S400T/K615R/K716N	G287S
2.2.1	A/Egypt/9174-NAMRU3/2009	H5N1	E1/E1	Human	2009-08-26	1	Female	M570I/K80R/T129N/I292M	T182I/K214R/L384S	N321S/S400T/K615R/K716N	G287S
2.2.1	A/Egypt/4822-NAMRU3/2009	H5N1	E1/E1	Human	2009-05-25	4	Female	D195N/M473V/T683I	T182I/K214R	N321S/K716N/L649I/A669V	T371M
2.2.1	A/Egypt/4979-NAMRU3/2009	H5N1	C1/C1	Human	2009-05-31	4	Female	D195N/M473V/T683I	T182I/K214R	N321S/K716N/L649I/A669V	T371M
2.2.1	A/Egypt/4935-NAMRU3/2009	H5N1	E1/E1	Human	2009-05-13	3	Male	M570I/K80R/T129N/I292M/I463M	T182I/K214R/L384S/K198R	N321S/S400T/K615R/K716N/E327K	G287S
2.2.1	A/Egypt/2752-NAMRU3/2009	H5N1	E1/E1	Human	2009-03-24	2	Female	M570I/K80R/T129N/Q257L	T182I/K214R/L384S/K198R	N321S/K716N	G287S
2.2.1	A/Egypt/2563-NAMRU3/2009	H5N1	C1/C1	Human	2009-03-14	38	Female	M570I/K80R/T129N/I292M	T182I/K214R/L384S/D175N	N321S/S400T/K615R/K716N	G287S
2.2.1	A/Egypt/3228-NAMRU3/2009	H5N1	E1	Human	2009-03-30	2	Male	M570I/K80R/T129N/I292M/I463M	T182I/K214R/L384S/K198R	N321S/S400T/K615R/K716N/E327K	G287S
2.2.1	A/Egypt/4396-NAMRU3/2009	H5N1	E1/E1	Human	2009-05-17	4	Female	M570I/K80R/T129N/I292M	T182I/K214R/L384S/K198R	N321S/S400T/K615R/K716N/V341L	G287S
2.2.1	A/Egypt/4394-NAMRU3/2009	H5N1	E1/E1	Human	2009-05-11	3	Male	M570I/K80R/T129N/I292M	T182I/K214R/L384S/A374T	N321S/S400T/K615R/K716N/V341L	G287S
2.2.1	A/Egypt/3450-NAMRU3/2009	H5N1	C1/C1	Human	2009-04-19	4	Male	M570I/K80R/T129N/Q257L	T182I/K214R/L384S/D175N/A374T	N321S/K716N/A669V	G287S
2.2.1	A/Egypt/N11981/2009	H5N1	E2/E1	Human	2009-11-22	3	Male	M570I/K80R/T129N/I292M/I463M	T182I/K214R/L384S/K198R	N321S/S400T/K615R/K716N	G287S
2.2.1	A/Egypt/N01360/2010	H5N1	C1/C1	Human	2010-02-02	40	Female	M570I/K80R/T129N/I292M/R299K	T182I/K214R/L384S/K198R	N321S/S400T/K615R/K716N/E382D/A669V	G287S
2.2.1	A/Egypt/N01644/2010	H5N1	E1/E1	Human	2010-02-07	37	Male	M570I/K80R/T129N/I292M	T182I/K214R/L384S/K198R	N321S/S400T/K615R/K716N/D55N/E382D	G287S
2.2.1	A/Egypt/N01982/2010	H5N1	E1/E1	Human	2010-02-12	30	Female	M570I/K80R/T129N/I292M	T182I/K214R/L384S	N321S/S400T/K615R/K716N	G287S
2.2.1	A/Egypt/N02038/2010	H5N1	E2/E1	Human	2010-02-15	10	Male	M570I/K80R/T129N/I292M	T182I/K214R/L384S/A374T	S400T/K615R/K716N/N321I	G287S
2.2.1	A/Egypt/N02127/2010	H5N1	E1/E1	Human	2010-02-13	30	Female	M570I/K80R/I292M	T182I/K214R/L384S	N321S/S400T/K615R/K716N	G287S
2.2.1	A/Egypt/N02554/2010	H5N1	E1/E2	Human	2010-02-23	1	Male	M570I/K80R/T129N/I292M	T182I/K214R/L384S	K615R/K716N/N321I	G287S
2.2.1	A/Egypt/N03071/2010	H5N1	E2/E1	Human	2010-03-03	1	Male	M570I/K80R/T129N	T182I/K214R/L384S	K615R/K716N/N321I	G287S
2.2.1	A/Egypt/N03072/2010	H5N1	E2/E1	Human	2010-03-07	20	Female	M570I/K80R/T129N/I292M/N456D	T182I/K214R/L384S/K198R	N321S/S400T/K615R/K716N/D55N/E382D	G287S
2.2.1	A/Egypt/N0544/2011	H5N1	E3	Human	2011	7	Male	M570I/K80R/T129N/I292M/R389K	T182I/K214R/L384S	N321S/S400T/K615R/K716N	G287S
2.2.1	A/Egypt/N6322/2011	H5N1	E2	Human	2011	4	Male	M570I/K80R/T129N/I292M/R389K	T182I/K214R/L384S	N321S/S400T/K615R/K716N	G287S
2.2.1	A/Egypt/N6658/2011	H5N1	E2	Human	2011	4	Male	M570I/K80R/T129N/I292M/R389K	T182I/K214R/L384S	N321S/S400T/K615R/K716N	G287S
2.2.1	A/Egypt/N0677/2011	H5N1	E2	Human	2011	1	Male	M570I/K80R/T129N/I292M	T182I/K214R/L384S	N321S/S400T/K615R/K716N	G287S
2.2.1	A/Egypt/N6774/2011	H5N1	E1	Human	2011	30	Female	M570I/K80R/T129N/I292M/R389K	T182I/K214R/L384S	N321S/S400T/K615R/K716N	G287S
2.2.1	A/Egypt/N6828/2011	H5N1	E3	Human	2011	1	Male	M570I/K80R/T129N/I292M	T182I/K214R/L384S	N321S/S400T/K615R/K716N	G287S
2.2.1	A/Egypt/N0423/2011	H5N1	E3	Human	2011	40	Female	M570I/K80R/T129N/I292M	T182I/K214R/L384S/K198R	N321S/S400T/K615R/K716N/D55N/E382D	G287S
2.2.1	A/Egypt/N7562/2011	H5N1	E3	Human	2011	21	Female	M570I/K80R/T129N/I292M	T182I/K214R/L384S	N321S/S400T/K615R	G287S
2.2.1	A/Egypt/N7592/2011	H5N1	E2	Human	2011	31	Male	M570I/K80R/T129N/I292M/R389K	T182I/K214R/L384S	N321S/S400T/K615R/K716N	G287S
2.2.1	A/Egypt/N7724/2011	H5N1	E2	Human	2011	27	Male	M570I/K80R/T129N/I292M/R389K	T182I/K214R/L384S	N321S/S400T/K615R/K716N	G287S
2.2.1	A/Egypt/N09966/2011	H5N1	E1	Human	2011-09-21	1	Male	M570I/K80R/T129N/I292M	T182I/K214R/L384S	N321S/S400T/K615R/K716N	G287S
2.2.1	A/Egypt/N11126/2011	H5N1	E1	Human	2011-12-01	21	Female	M570I/K80R/T129N/I292M/T637I	T182I/K214R/L384S/K54R/A374T	N321S/S400T/K615R/K716N	G287S
2.2.1	A/Egypt/N11470/2011	H5N1	E1	Human	2011-12-15	29	Male	M570I/K80R/T129N/I292M/T637I	T182I/K214R/L384S/K54R/A374T	N321S/S400T/K615R/K716N	G287S
2.2.1	A/Egypt/N14976/2011	H5N1	E2	Human	2011-12-21	42	Male	M570I/K80R/T129N/I292M/T637I	T182I/K214R/L384S/K54R/A374T	N321S/S400T/K615R/K716N	G287S
2.2.1	A/Egypt/N10621/2011	H5N1	E1	Human	2011-10-30	2	Female	M570I/T129N/I292M	T182I/K214R/L384S	N321S/S400T/K615R/K716N	G287S

[†]C, cultured cells; E, eggs

[‡]*U*, unkown

[*]Mutations were identified by comparing each gene sequence to the clade 2.2.1 consensus sequence.
