# Supplementary material for: Novel Polymerase Gene Mutations for Human Adaptation in Clinical Isolates of Avian H5N1 Influenza Viruses
Source: PLoS Pathog. 2016 Apr 20;12(4):e1005583. doi: 10.1371/journal.ppat.1005583 (PMC4838241; doi:10.1371/journal.ppat.1005583)
Supplement: S2 Table — (PDF) [file ppat.1005583.s015.pdf]

**S2 Table. Prevalence of the clade 2.2.1 virus polymerase and NP mutations identified in the database search in this study.**

| | | | % of strains with mutation (no. of strains) | |
|---|---|---|---|---|
| Gene (no. of mutations) | Category of mutation | Mutation | Human viruses[a] | Bird viruses[a] |
| PB2 (14) | Only in human viruses | Q257L | 4.9 (3) | 0 (0) |
| | | R299K | 4.9 (3) | 0 (0) |
| | | N456D | 4.9 (3) | 0 (0) |
| | | I463M | 8.2 (5) | 0 (0) |
| | | K617E | 4.9 (3) | 0 (0) |
| | | T637I | 4.9 (3) | 0 (0) |
| | | T683I | 4.9 (3) | 0 (0) |
| | More prevalent in human viruses than in avian viruses | K80R | 68.9 (42) | 20.9 (19) |
| | | T129N | 67.2 (41) | 20.9 (19) |
| | | D195N | 8.2 (5) | 1.1 (1) |
| | | I292M | 59.0 (36) | 20.9 (19) |
| | | R389K | 9.8 (6) | 1.1 (1) |
| | | M473V | 13.1 (8) | 3.3 (3) |
| | | M570I | 72.1 (44) | 47.3 (43) |
| PB1 (13) | Only in human viruses | K54R | 4.9 (3) | 0 (0) |
| | | D175N | 4.9 (3) | 0 (0) |
| | | K198R | 23.0 (14) | 0 (0) |
| | | A374T | 9.8 (6) | 0 (0) |
| | | M616R | 11.5 (7) | 0 (0) |
| | | E618D | 8.2 (5) | 0 (0) |
| | | P627L | 11.5 (7) | 0 (0) |
| | | N642K | 11.5 (7) | 0 (0) |
| | | P756S | 9.8 (6) | 0 (0) |
| | More prevalent in human viruses than in avian viruses | T182I | 85.2 (52) | 28.2 (22) |
| | | K214R | 85.2 (52) | 28.2 (22) |
| | | L384S | 65.5 (40) | 24.4 (19) |
| | | K386R | 6.6 (4) | 1.3 (1) |
| PA (17) | Only in human viruses | D55N | 8.2 (5) | 0 (0) |
| | | N321I | 4.9 (3) | 0 (0) |
| | | E327K | 6.6 (4) | 0 (0) |
| | | V341L | 4.9 (3) | 0 (0) |
| | | E382D | 6.6 (4) | 0 (0) |
| | | T608S | 4.9 (3) | 0 (0) |
| | | E629H | 9.8 (6) | 0 (0) |
| | | G631C | 11.5 (7) | 0 (0) |
| | | L641P | 11.5 (7) | 0 (0) |
| | | S644F | 8.2 (5) | 0 (0) |
| | | L649I | 4.9 (3) | 0 (0) |
| | | A669V | 11.5 (7) | 0 (0) |
| | | F681L | 9.8 (6) | 0 (0) |
| | More prevalent in human viruses than in avian viruses | N321S | 80.3 (49) | 29.5 (23) |
| | | S400T | 57.4 (35) | 25.6 (20) |
| | | K615R | 60.7 (37) | 24.4 (19) |
| | | K716N | 67.2 (41) | 28.2 (22) |
| NP (2) | More prevalent in human viruses than in avian viruses | G287S | 73.8 (45) | 24.7 (20) |
| | | T371M | 16.4 (10) | 2.5 (2) |

[a] For human viruses there were 61 PB2, PB1, PA and NP sequences, and for avian viruses there were 91 PB2, 78 PB1, 78 PA and 81 NP sequences.
