# Supplementary material for: Novel Polymerase Gene Mutations for Human Adaptation in Clinical Isolates of Avian H5N1 Influenza Viruses
Source: PLoS Pathog. 2016 Apr 20;12(4):e1005583. doi: 10.1371/journal.ppat.1005583 (PMC4838241; doi:10.1371/journal.ppat.1005583)
Supplement: S3 Table — (PDF) [file ppat.1005583.s016.pdf]

S3 Table. Summary of the results from various assays in this study.

Serial No.	Type of mutation	Gene (No. of mutations)	Mutation	Inter-segment combination with mutation	In vitro polymerase activity in: †						In vitro virus growth in: ‡			In vivo virus titers in: *	
					293T cells transfected with				QT-6 cells transfected with		SAE cells at		CEF cells at	Mouse lungs at	
					vRNA at 37 °C	vRNA at 33 °C	cRNA at 37 °C	cRNA at 33 °C	vRNA at 37 °C	cRNA at 37 °C	37°C	33°C	37°C	3 d post-infection	6 d post-infection
1	Single mutations	PB2 (14)	K80R		=	=	=	+	=	=	ND[‡]	ND	ND	ND	ND
2			T129N		−	−	−	−	−	−	ND	ND	ND	ND	ND
3			D195N		=	+	+	+	=	=	ND	ND	ND	ND	ND
4			Q257L		−	−	−	−	−	−	ND	ND	ND	ND	ND
5			I292M		−	−	−	−	=	=	ND	ND	ND	ND	ND
6			R299K	Inter-5	=	=	=	=	=	=	=	−	−	ND	ND
7			R389K	Inter-8	+	+	+	+	+	=	=	−	−	ND	ND
8			N456D		+	=	+	+	=	=	ND	ND	ND	ND	ND
9			I463M	Inter-3	−	=	−	=	−	=	=	−	−	ND	ND
10			M473V		=	−	=	=	=	=	ND	ND	ND	ND	ND
11			M570I		=	=	=	=	=	=	ND	ND	ND	ND	ND
12			K617E		−	−	−	−	−	−	ND	ND	ND	ND	ND
13			T637I		−	−	−	−	−	−	ND	ND	ND	ND	ND
14			T683I		−	−	−	−	−	−	ND	ND	ND	ND	ND
15		PB1 (13)	K54R		=	=	+	+	=	=	ND	ND	ND	ND	ND
16			D175N		−	=	−	=	−	−	ND	ND	ND	ND	ND
17			T182I		=	−	++	=	=	+	+	++	=	ND	ND
18			K198R	Inter-3,4,5	+	=	+++	++	=	+	+	+++	+	+	++
19			K214R		+	=	=	+	=	=	ND	ND	ND	ND	ND
20			A374T		−	=	−	=	−	−	ND	ND	ND	ND	ND
21			L384S		=	=	+	=	=	+	ND	ND	ND	ND	ND
22			K386R		=	=	+	=	−	=	ND	ND	ND	ND	ND
23			M616R		−	−	−	−	−	−	ND	ND	ND	ND	ND
24			E618D		−	−	=	=	−	=	ND	ND	ND	ND	ND
25			P627L		=	+++	=	+++	−	=	=	++	=	ND	ND
26			N642K		−	−	−	−	−	−	ND	ND	ND	ND	ND
27			P756S		−	−	=	=	−	=	ND	ND	ND	ND	ND
28		PA (17)	D55N		−	−	−	−	−	−	ND	ND	ND	ND	ND
29			N321S		−	=	=	=	=	=	ND	ND	ND	ND	ND
30			N321I	Inter-6	=	=	=	=	=	=	−	=	−	ND	ND
31			E327K	Inter-3	=	+	+	++	=	=	−	−	=	ND	ND
32			V341L	Inter-4	=	=	=	+	=	=	−	−	=	ND	ND
33			E382D		−	=	−	=	−	−	ND	ND	ND	ND	ND
34			S400T		=	=	=	=	−	=	ND	ND	ND	ND	ND
35			T608S		−	−	−	−	−	−	ND	ND	ND	ND	ND
36			K615R		−	−	−	=	−	=	ND	ND	ND	ND	ND
37			E629H		−	−	−	−	−	−	ND	ND	ND	ND	ND
38			G631C		−	−	=	−	=	=	ND	ND	ND	ND	ND
39			L641P		−	−	−	−	−	−	ND	ND	ND	ND	ND
40			S644F		−	−	−	−	−	−	ND	ND	ND	ND	ND
41			L649I		=	+	=	=	=	=	ND	ND	ND	ND	ND
42			A669V		=	+	+	=	=	=	ND	ND	ND	ND	ND
43			F681L		−	−	−	−	−	−	ND	ND	ND	ND	ND
44			K716N		=	=	=	=	−	=	ND	ND	ND	ND	ND
45		NP (2)	G287S	Inter-1,3,4,5,6,8	=	=	=	=	=	=	=	=	=	ND	ND
46			T371M		=	=	=	=	=	=	ND	ND	ND	ND	ND
47	Intra-segment combinations	PB2(8)	D195N/R299K		=	+	+	+	=	=	ND	ND	ND	ND	ND
48			D195N/R299K/N456D		+	+	++	++	=	=	=	=	=	ND	ND
49			M473V/D195N	Inter-2,7	=	=	=	+	=	=	+	+	=	ND	ND
50			M473V/D195N/T683I		−	−	−	=	−	=	ND	ND	ND	ND	ND
51			M473V/N456D		=	=	+	+	=	=	ND	ND	ND	ND	ND
52			M570I/K80R	Inter-1,4,6	+	=	+	+	=	=	=	=	=	ND	ND
53			M570I/K80R/T129N		−	−	−	−	−	−	ND	ND	ND	ND	ND
54			M570I/K80R/T129N/I292M		−	−	−	−	−	−	ND	ND	ND	ND	ND
55		PB1(7)	T182I/K214R	Inter-2,7	+	−	++	+	=	=	=	=	=	ND	ND
56			T182I/K214R/L384S	Inter-6,8	+	=	+++	+	+	+	+	=	=	ND	ND
57			K198R/D175N	Inter-1	+	=	+++	++	−	=	++	++	+	+++	+++
58			A374T/K54R		−	−	−	=	−	−	ND	ND	ND	ND	ND
59			P756S/P627L		+	+++	+	++	=	=	ND	ND	ND	ND	ND
60			P756S/P627L/E618D		+	+++	+	+++	=	=	ND	ND	ND	ND	ND
61			P756S/P627L/E618D/K386R		++	+++	++	++	=	=	−	−	=	ND	ND
62		PA(7)	N321S/K716N	Inter-7	=	=	−	=	−	−	−	−	=	ND	ND
63			N321S/K716N/S400T	Inter-8	−	−	−	−	−	−	ND	ND	ND	ND	ND
64			N321S/K716N/S400T/K615R		−	−	−	−	−	−	ND	ND	ND	ND	ND
65			G631C/E629H		−	−	−	−	−	−	ND	ND	ND	ND	ND
66			G631C/E629H/A669V		−	−	−	−	−	−	ND	ND	ND	ND	ND
67			A669V/L649I	Inter-2	=	=	+	=	=	−	−	−	−	ND	ND
68			A669V/E382D	Inter-5	−	−	−	−	−	−	−	−	−	ND	ND
69		NP(1)	G287S/T371M	Inter-2,7	=	=	=	=	=	=	=	=	−	ND	ND
70	Inter-segment combination of mutations	Intersegment(16)	Inter-1		+	+	+++	+++	+	+	++	+++	+	ND	ND
71			Inter-2		+	+	+++	+++	=	=	++	+++	=	+++	++
72			Inter-3		+	+	+++	+++	+	=	=	++	=	ND	ND
73			Inter-4		+	+	+++	+++	+	+	++	+++	=	+++	+++
74			Inter-5		+	+	+++	++	+	=	−	=	=	ND	ND
75			Inter-6		+	+	+++	+++	++	+	−	−	=	ND	ND
76			Inter-7		+	=	++	+++	+	=	−	=	=	ND	ND
77			Inter-8		+	+	+++	++	++	+	−	−	=	ND	ND
78			Inter-9		−	−	=	−	−	−	ND	ND	ND	ND	ND
79			Inter-10		+	=	++	++	+	=	ND	ND	ND	ND	ND
80			Inter-11		+	=	++	++	+	+	ND	ND	ND	ND	ND
81			Inter-12		−	=	−	=	−	−	ND	ND	ND	ND	ND
82			Inter-13		+	+	++	++	=	=	ND	ND	ND	ND	ND
83			Inter-14		−	−	+	=	−	=	ND	ND	ND	ND	ND
84			Inter-15		−	=	−	=	−	−	ND	ND	ND	ND	ND
85			Inter-16		−	+	−	+	−	−	ND	ND	ND	ND	ND

[†]Relative comparison with wild-type polymerase activity: +, 1.5-3-fold increase; ++, 3-5-fold increase; +++, >5-fold increase; =, similar to wild-type virus (<1.5-fold change); −, lower than wild-type.

[‡]Relative comparison with wild-type virus yield: +, 2-5-fold increase; ++, >5-10-fold increase; +++, >10-fold increase; =, similar to wild-type virus (<2-fold change); −, lower that wild-type.

[*]Relative comparison with wild-type virus titer: +, 2-10-fold increase; ++, >10-100-fold increase; +++, >100-fold increase; =, similar to wild-type virus (<2-fold change); −, lower that wild-type.

[‡]ND: not determined.
