# Supplementary material for: Novel Polymerase Gene Mutations for Human Adaptation in Clinical Isolates of Avian H5N1 Influenza Viruses
Source: PLoS Pathog. 2016 Apr 20;12(4):e1005583. doi: 10.1371/journal.ppat.1005583 (PMC4838241; doi:10.1371/journal.ppat.1005583)
Supplement: S4 Table — (PDF) [file ppat.1005583.s017.pdf]

S4 Table. Interaction energy bewteen the vRNA promoter and vRNA contact residues in EG/D1 (wt) PB1 and PB1 mutants.

Mutation	IntE (kcal/mol) between vRNA promoter and vRNA contact residues in PB1 β-ribbon (ΔE)[a]		IntE (kcal/mol) between vRNA promoter and trimeric polymerase complex(ΔE)[b]
	188	203	
EG/D1 (Wt)	-85.71	-48.73	-1098.29
PB1-T182I	-51.92 (33.79)	-35.21 (13.52)	-1230.78 (-132.49)
PB1-K214R	-56.53 (29.18)	-27.37 (21.36)	-1175.62 (-77.33)

[a]The interaction energy, IntE, was calculated between the vRNA promoter and the PB1 contact residues for the EG/D1 (wt) and mutant polymerases. ΔE was the IntE difference between the mutant and EG/D1 residues.

[b]IntE was calculated between the vRNA promoter and the trimeric polymerase complexes for EG/D1 (wt) and the PB1 mutants. ΔE was the IntE difference between the mutant and EG/D1 trimeric polymerase complexes.
